# xCT contributes to colorectal cancer tumorigenesis through upregulation of the MELK oncogene and activation of the AKT/mTOR cascade

**DOI:** 10.1038/s41419-022-04827-4

**Published:** 2022-04-19

**Authors:** Bufu Tang, Jinyu Zhu, Fangming Liu, Jiayi Ding, Yajie Wang, Shiji Fang, Liyun zheng, Rongfang Qiu, Minjiang Chen, Gaofeng Shu, Min Xu, Chenying Lu, Zhongwei Zhao, Yang Yang, Jiansong Ji

**Affiliations:** 1 Key Laboratory of Imaging Diagnosis and Minimally Invasive Intervention Research, Lishui Hospital, School of Medicine, Zhejiang University, Lishui, 323000 China; 2 Department of Radiology, Sir Run Run Shaw Hospital, School of Medicine, Zhejiang University, Hangzhou, 310016 China; 3 Department of Radiology, Second Affiliated Hospital, School of Medicine, Zhejiang University, Hangzhou, China; 4 Chinese Academy of Medical Sciences and Peking Union Medical College Institute of Basic Medical Sciences, Beijing, 10005 China; 5 Interventional treatment center, the Fifth Affiliated Hospital of Wenzhou Medical University, Lishui, 323000 China

**Keywords:** Oncogenes, Cancer stem cells, Tumour biomarkers

## Abstract

Colorectal cancer (CRC) is one of the most commonly diagnosed and deadly malignant tumors globally, and its occurrence and progression are closely related to the poor histological features and complex molecular characteristics among patients. It is urgent to identify specific biomarkers for effective treatment of CRC. In this study, we performed comprehensive experiments to validate the role of xCT expression in CRC tumorigenesis and stemness and confirmed xCT knockdown significantly suppressed the proliferation, migration, and stemness of CRC cells in vitro and effectively inhibited CRC tumorigenesis and metastasis in vivo. In addition, bioinformatic analysis and luciferase assays were used to identify E2F1 as a critical upstream transcription factor of SLC7A11 (the gene encoding for xCT) that facilitated CRC progression and cell stemness. Subsequent RNA sequencing, western blotting, rescue assay, and immunofluorescence assays revealed MELK directly co-expressed with xCT in CRC cells, and its upregulation significantly attenuated E2F1/xCT-mediated tumorigenesis and stemness in CRC. Further molecular mechanism exploration confirmed that xCT knockdown may exert an antitumor effect by controlling the activation of MELK-mediated Akt/mTOR signaling. Erastin, a specific inhibitor of xCT, was also proven to effectively inhibit CRC tumorigenesis and cell stemness. Altogether, our study showed that E2F1/xCT is a promising therapeutic target of CRC that promotes tumorigenesis and cell stemness. Erastin is also an effective antitumoral agent for CRC.

## Introduction

Colorectal cancer (CRC) is the sixth most commonly diagnosed malignant tumor and the third most deadly cancer globally[1]; it accounts for approximately 9.4% of all cancer-related deaths worldwide in 2020, according to the WHO statistics (https://gco.iarc.fr/today/fact-sheets-cancers). It was found that occurrence and progression of CRC are closely related to its unique and complex molecular characteristics [2, 3]. Exploring the molecular characteristics of CRC patients may be beneficial to help effectively prevent and diagnose CRC and provide more options for targeted therapy.

xCT, which is encoded by solute carrier family 7 member 11 (SLC7A11), is a cystine/glutamate antitransporter that mediates the biosynthesis of antioxidant glutathione and antioxidant defense [4], which is essential for the survival of tumor cells and participates in mediating the growth and malignant progression of tumor cells [5, 6]. In recent years, it was found that xCT molecules play an important role in tumor growth, progression, metastasis, and multidrug resistance in various types of cancer [7, 8]. High xCT activity serves as a predictor of disease recurrence in patients with CRC [9]. However, the role of xCT in tumorigenesis of CRC and the underlying molecular mechanism of xCT in CRC progression need to be further elucidated.

Maternal embryonic leucine zipper kinase (MELK) is a member of the AMPK/Snf1 family, and its encoded serine/threonine kinase is involved in various physiological and pathological processes, such as cell cycle regulation, cell proliferation, apoptosis, embryonic development, and tumor development [10, 11]. In recent years, many studies have reported that MELK has potential carcinogenic effects and plays a key role in maintaining the stemness of cancer cells [12]. It is significantly overexpressed in many cancers, such as CRC, lung cancer, and liver cancer, while MELK silencing slows or inhibits the growth, invasion, stemness, and tumorigenicity of these cancers and other cancer cell lines [13–15]. However, the regulatory mechanism of MELK in the tumorigenesis of CRC is still unclear.

In this study, we analyzed the effects of xCT expression on the proliferation, migration, and tumorigenesis of CRC cells in vitro and in vivo and evaluated the antitumor effects of the xCT inhibitor erastin in CRC. We also explored the corresponding molecular mechanism by assessing factors including upstream regulatory transcription factors and downstream essential molecules and signaling networks. This study comprehensively explored the oncogenic role of xCT in the tumorigenesis and progression of CRC, and our research may help open up new ideas and options for targeted therapy for CRC.

## Materials and methods

### Antibodies

Antibodies against xCT (ab37185, WB:1:1000, IF: 1:200), CD133 (ab216323, Flow Cyt: 1:100), MELK (ab273015, WB:1:1000, IF: 1:200), S6K (ab32529, WB:1:1000), P-S6K (ab131436, WB:1:1000), β-Actin (ab8226, WB:1:4000) were purchased from Abcam (Cambridge, UK). Antibodies against PCNA (#13110, WB:1:1000, IF: 1:400), Vimentin (#5741, WB:1:1000, IF: 1:200), N-cadherin (#13116, WB:1:1000, IF: 1:200, IHC: 1:200), CD133 (#64326, WB:1:1000, IF: 1:200, IHC: 1:200), E-cadherin (#3195, IHC: 1:400), Ki67 (#9027, IHC: 1:400), E2F1 (#3742, WB:1:1000), P-AKT (#4060, WB:1:1000, IHC: 1:100), C-myc (#18583, WB:1:1000), MMP1 (#54376, WB:1:1000), EPCAM (#93790, WB:1:1000), LIN28 (#3695, WB:1:1000), SOX2 (#3579, WB:1:1000), mTOR (#2983, WB:1:1000), P-mTOR (#5536, WB:1:1000), AKT (#4691, WB:1:1000), 4EBP1 (#9644, WB:1:1000), P-4EBP1 (#2855, WB:1:1000) were obtained from Cell Signaling Technology, Inc. (Beverly, MA). OCT3/4 (sc-365509, WB: 1:500) was purchased from Santa Cruz Biotechnology (CA, USA). Secondary antibodies, including Goat anti-rabbit IgG HRP (#7074, WB: 1:15000) and Goat anti-mouse IgG HRP (#7076, WB: 1:15000) were purchased from Cell Signaling Technology.

### Data collection

The mRNA data and corresponding clinical information of CRC patients (including 41 COAD tumor tissues and paired paracancer tissues, and 9 READ tumor tissues and paired paracancer tissues) used in the study were mainly obtained from The Cancer Genome Atlas (TCGA) database (https://www.cancer.gov/about-nci/organization/ccg/research/structural-genomics/tcga) and the GSE87211, GSE17538 and GSE39582 datasets from the Gene Expression Omnibus (GEO) database (https://www.ncbi.nlm.nih.gov/geo/). In addition, we collected 49 CRC samples and the matched 65 normal samples from patients diagnosed with CRC and underwent resection at the Lishui Hospital of Zhejiang University between 2019 and 2021 with corresponding clinical information (including age, sex, clinicopathological characteristics, overall survival time and survival status) as a validation cohort. The study was approved by the Institutional Review Committee of Lishui Hospital of Zhejiang University and all patients provided written informed consent.

### Cell culture, cell transfection, and cell lentivirus infection

Human CRC cell lines (including HCT116 and HCT15) were purchased from the American Type Culture Collection (Manassas, VA, USA) and cultured in DMEM containing 2 mM L-glutamine, 100 U/mL penicillin-streptomycin, and 10% fetal bovine serum. The cells were incubated in a constant temperature incubator at 37 °C with an atmosphere containing 5% CO2 and 95% relative humidity. The cells were cytogenetically tested and authenticated before being frozen. The xCT-siRNA, lenti-scramble and lenti-xCT-shRNA viruses were generated from GenePharma (Shanghai, China). Lipofectamine 3000 transfection reagent (Invitrogen, Carlsbad, CA, USA) was used for siRNA transient transfection according to the manufacturer’s protocol. The cells were cultured in a six-well plate then added lenti-scramble and lenti-xCT-shRNA viruses according to the manufacturer’s protocol. After 72 h of shRNA lentivirus infection, the infected cells were cultured in DMEM containing 1–2 ng/ml puromycin, 2 mM L-glutamine, 100 U/mL penicillin-streptomycin and 10% fetal bovine serum for 7–10 days. Then the stably infected cells are seeded in a six-well plate at a ratio of 1:10, single cells are visible in the six-well plate and divide and multiply to form single cell colonies. The cell colonies were harvested using 0.25% trypsin and a continuous 10-fold dilution [10^−2^–10^−10^] were made and each dilution of cells were added to the 96-well plate for culture. After 7–10 days, the expression of xCT in monoclonal cells was detected by Western blot, and the clone with the lowest expression was selected for culture and subsequent experiments. Details of the siRNA and shRNA sequences are listed in the supporting information.

### Cell cycle assay

HCT116 and HCT15 cells were fixed in 70% cold ethanol for 24 h and treated with the Cell Cycle Assay Kit (Beyotime, Shanghai, China) according to the manufacturer’s instructions. A BD FACSCanto II^TM^ flow cytometer (Lake Franklin, NJ, USA) was used to analyze the cell cycle.

### Cell proliferation assay

The Cell Counting Kit-8 (CCK-8) assay and 5-ethynyl-2′-deoxyuridine (EdU) assay were performed to measure proliferation of CRC cell lines. HCT116 and HCT15 cells were seeded into a 96-well plate at 3000 cells per well, and 100 μM DMEM was added to each well. After incubation of the cells in a constant temperature incubator at 37 °C with an atmosphere of 5% CO2 and 95% air for 0, 24, 48, and 72 h, 20 μL CCK-8 reagent was added to each well according to the manufacturer’s instructions, and the incubation was continued at 37 °C for 2 h. A microplate reader (Bio-Rad, Berkeley, USA) was used to detect the absorbance of the cells at 450 nm. Each group had three replicate wells. An EdU assay was performed using an EdU agent obtained from Ruibo Company (Guangzhou, China) following the manufacturer’s protocol to measure cell proliferation.

### Colony formation assay

HCT116 and HCT15 cells were harvested using 0.25% trypsin, pipetted to break up cell clumps, then suspended in DMEM and placed in a six-well plate at a density of 50 cells per well. After 3 weeks of culture in a constant temperature incubator, the cells were fixed with 4% paraformaldehyde and stained with 0.1% crystal violet. The relative number of colonies was counted under a microscope.

### Transwell assay

Transwell assay was carried out to measure cell migration ability. Approximately 2 × 10^5^ CRC cells (including HCT116 and HCT15 cells) were suspended in 200 μl serum-free DMEM containing 0.1% BSA and added to the upper chamber of a 24-well culture plate equipped with a 8.0 μm pore size polycarbonate membrane. DMEM supplemented with 10% fetal bovine serum was added to the lower chamber. After incubation of cells at 37 °C for 72 h, the cells passing through the membrane were stained with 0.4% trypan blue and counted under an optical microscope. To exclude the influence of proliferation on migration, CRC cells were treated with mitomycin C (10 μg/ml) for 1 h to inhibit cell proliferation prior to the transwell assay. Each experiment was repeated three times independently.

### Wound scratch assay

On the back of a six-well plate, we drew straight lines across the hole every 0.5–1 cm. CRC cells were suspended in DMEM containing 10% fetal bovine serum, and approximately 1 × 10^6^ cells were added to each well and incubated in an incubator at 37 °C overnight. Then, a 10 µl pipette tip was used to draw two parallel lines in cells perpendicular to the marking lines. The cells were washed gently with PBS 3 times to remove the floating cells, added to serum-free DMEM medium and incubated in an incubator at 37 °C for 48 h.To prevent the influence of cell proliferation on migration, cells were preincubated with mitomycin C (10 μg/ml) for 1 h before the wound scratch assay. A microscope was used to observe and photograph cells near the scratch.

### CSCs sorting

HCT116 and HCT15 cells were cultured in DMEM/F12 medium supplemented with EGF (20 ng/mL, Sigma, USA), basic fibroblast growth factor (bFGF) (20 ng/mL, Sigma, USA) and B27 (1X, GiBCO) in 6-well plates with ultralow-adhesion surfaces (Corning Incorporated) until spheroids were formed. Then the formed spheroids were resuspended in DMEM/F12 medium to obtain the single-cell suspension, and the CD44-positive cells in the cell suspension were sorted out using the BD FACSAria Cell Sorter (P07900037), which were the CSCs of the CRC.

### Immunofluorescence (IF) staining

HCT116 and HCT15 cells were cultured on glass coverslips attached to a six-well plate. Then, 4% paraformaldehyde was used to fix the cells, and 0.3% Triton X-100 was used to enhance the permeability of the cell membranes. Then, the cells were incubated in 5% bovine serum albumin for 1 h and incubated in the corresponding primary antibody at 4 °C overnight, followed by incubation with the secondary antibody at room temperature for another 2 h. DAPI (300 nM) staining was employed for nuclear localization. The fluorescence staining of the cells was visualized using a fluorescence microscope (Nikon, Japan). The fluorescent images were acquired using CaseViewer software (version 2.3) (3DHISTECH, Budapest, Hungary) and analyzed with ImageJ software (version 1.8.0).

### Nude mouse xenograft assay

Male BALB/c nude mice aged 4-6 weeks were purchased from Shanghai Slack Laboratory Animal Co., Ltd. (Shanghai, China) for the construction of CRC xenograft mouse models. The nude mice were randomly divided into two groups (6 mice in each group), and CRC cells were resuspended in PBS and injected subcutaneously into the sides of the lower abdomen of nude mice at 3 × 10^6^ cells. One group was injected with HCT116 cells, and the other group was injected with HCT116 cells transfected with lenti-xCT-shRNA. When the subcutaneous tumors became visible to the naked eye (approximately 2 mm at approximately 1 week), the mice received intraperitoneal injections of 40 mg/kg erastin or vehicle control (saline) at the same concentration every other day. After approximately 3 weeks, the nude mice were euthanized by cervical dislocation under isoflurane anesthesia, and the tumors were removed for observation. The tumor volume (TV) was calculated according to the following formula: TV (mm^3^) = *L* × *W* ^2^ × 0.5.

### CRC lung metastasis mouse model construction

Male BALB/c nude mice aged 4–6 weeks were used to construct CRC lung metastasis mouse models. The nude mice were randomly divided into two groups (6 mice in each group), and CT26 cells were resuspended in PBS and intravenously injected into the tail vein of mice as slowly as possible with approximately 1 × 10^6^ cells/mouse. One group was injected with CT26 infected with lenti-scramble cells, and the other group was injected with CT26 cells infected with lenti-xCT-shRNA. 3 weeks after the injection, mice were euthanized by cervical dislocation under isoflurane anesthesia, and the lung was removed for observation and HE staining.

### Immunohistochemical (IHC) staining

The tumors isolated from the nude mice treated with or without erastin were fixed in 4% paraformaldehyde for 24 h and embedded in paraffin. Then, the tumor tissues were sectioned (4 × 4 μm), and the paraffin sections were deparaffinized twice using xylene (5–10 min/wash), followed by rehydration in absolute ethanol for 5 min, 90% ethanol for 2 min, 70% ethanol for 2 min and distilled water for 2 min. The endogenous peroxidase activity in the sectioned tissues was eliminated using methanol containing 30% H_2_O_2_, and the antigen was retrieved by heat induction. Then, the sections were blocked in PBS containing 10% fetal bovine serum for 45 min and incubated in the primary antibody at 4 °C overnight, followed by another incubation with the secondary antibody conjugated with horseradish peroxidase for another 2 h at room temperature. 3,3′-Diaminobenzidine (DAB) was applied to stain the tissues.

### Western blot analysis

RIPA lysis buffer supplemented with phenylmethylsulfonyl fluoride (PMSF) at a concentration of 1 mM was used to lyse HCT116 and HCT15 cells to obtain the corresponding proteins. The protein samples were separated using 10–15% sodium dodecyl sulfate-polyacrylamide gel electrophoresis (SDS-PAGE) and electrotransferred to a polyvinylidene fluoride (PVDF) membrane. Then, the membrane was blocked in 5% skim milk for 90 min in a shaker at room temperature, followed by incubation with the primary antibody in a shaker at 4 °C overnight. Subsequently, the membrane was washed with Tris-buffered saline with Tween (TBST) three times and incubated with the secondary antibody for another 2 h. The bands on the membrane were detected and imaged using the iBright™ FL1500 Imaging System.

### Flow cytometry

Flow cytometry was performed to detect changes in the expression of stemness-related markers (including EPCAM and CD133) in HCT116 and HCT15 cells. A minimum of 20,000 cells were counted and analyzed under each condition using a BD FACSCanto II^TM^ flow cytometer (Lake Franklin, NJ, USA), and the data were further analyzed using FlowJo software (Version 10.0). Each experiment was independently repeated at least 3 times.

### Dual-luciferase reporter gene assay

The CDS region of SLC7A11 was searched through NCBI and the sequence of 2000bp and 5′UTR (280 bp) from the ATG upstream of the CDS region was looked for as the promoter region of SLC7A11, and the potential binding sites of the E2F1 to SLC7A11 promoter regions were predicted through The JASPAR database (https://jaspar.genereg.net/). The nucleotide containing protomor of SLC7A11 (from -2000 to 100 of human SLC7A11 gene loci) was used to predict the potential transcription factor binding sites, and nucleotide containing wild-type (WT) SLC7A11 promoter fragment or binding site mutant (MUT) SLC7A11 3′-UTR (SLC7A11-WT, SLC7A11-MUT1, SLC7A11-MUT2, and SLC7A11-MUT3) were cloned into pGL4.10 vector by GenePharma (Shanghai, China). Then the vectors were co-transfected with E2F1 into cells by the Lipofectamine 3000 transfection reagent for 48 h. Later, the cells were harvested and lysed and firefly luciferase activity was determined using a Dual-Glo luciferase assay kit (Promega, Madison, WI, USA).

### Quantitative reverse transcription-polymerase chain reaction (qRT-PCR)

TRIzol^®^ reagent was applied to extract the total RNA from cultured HCT116 and HCT15 cells, and the RevertAid First Strand cDNA Synthesis kit was used to reverse transcribe the extracted total RNA into cDNA according to the manufacturer’s protocol, followed by qRT-PCR using the SuperScript III Platinum SYBR Green One-Step qRT-PCR Kit with ROX. The 2-ΔΔCT method was used for the determination of the relative expression (fold change) of the target genes. β-Actin served as an internal control. Each experiment was repeated three times independently. The primer sequences used for the qRT-PCR are listed in the supporting information.

### Calculation of gene expression‐based stemness indices (such as the mRNA stemness index [mRNAsi]) for CRC

Stemness indices are indicators that describe the similarity of tumor cells to stem cells, and a gene expression-based stemness index (mRNAsi) can be considered a quantification of CSCs, with a range of 0–1; the closer the value is to 1, the lower the cell differentiation and the stronger the stem cell characteristics of the corresponding sample [16]. In this study, we calculated the mRNAsi of patients with colon adenocarcinoma (COAD) in the TCGA database through a predictive model, which was constructed by Malta et al. [17] using a one-class logistic regression (OCLR) algorithm on pluripotent stem cell samples from the Progenitor Cell Biology Consortium (PCBC) dataset (https://progenitorcells.org/frontpage) [18]. The PanCanStem Web tool (https://bioinformaticsfmrp.github.io/PanCanStem_Web/) was used to determine mRNAsi values. We adopted the stemness index predictive model to score patients with COAD using the Spearman correlation operator and mapped mRNAsi ranging from 0 to 1 by utilizing a linear transformation that subtracted the minimum and divided by the maximum [16].

### Identification of differentially expressed genes (DEGs) in HCT15 cells with or without xCT inhibition

The mRNA sequence obtained from HCT15 cells with or without xCT knockdown was used for differential expression analysis. DEGs were identified using the limma R package with cutoff values of absolute log 2-fold change (FC) > 1 and adjusted *P* value < 0.05.

### Functional enrichment analysis

Kyoto Encyclopedia of Genes and Genomes (KEGG) pathway enrichment analysis of the DEGs was performed by employing the KOBAS database (kobas.cbi.pku.edu.cn). *P* < 0.05 was considered to represent statistical significance.

### Statistical analysis

Statistical difference analysis of the experimental data was conducted via SPSS (version 24.0) and GraphPad Prism (version 8.0) software. The difference between the two groups was validated by Student’s t test, and the difference among multiple groups was compared by one-way ANOVA and Bonferroni test. The data are presented as the mean ± SD or SEM. *P* < 0.05 was considered to indicate statistical significance. All experiments were repeated at least three times independently.

## Results

### xCT is highly expressed in patients with primary CRC and predicts a poor prognosis

**Fig. 1:**
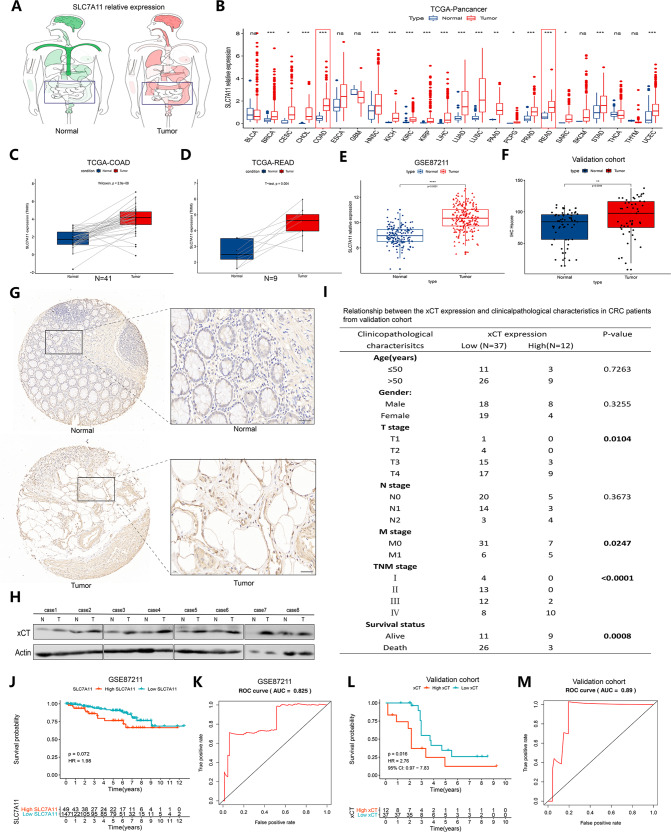
xCT is highly expressed in patients with primary CRC and predicts a poor prognosis. **A** Differences in the expression of xCT between tumors and normal tissues of various organs were observed in the TCGA database. **B** An overall exploration of the expression characteristics of xCT in the TCGA pancancer dataset. **C**, **D** Difference in the expression of xCT between patients with COAD or READ and matched normal samples in the TCGA database. **E** Difference in the expression of xCT between 203 CRC samples and 160 mucosa control samples in the GSE87211 dataset. **F** Difference in the expression of xCT between CRC samples and matched normal samples from our collected 49 CRC subjects was assessed using IHC staining. **G** IHC staining showed that xCT was more positively expressed in CRC tissues than in normal tissues from our collected CRC samples. **H** Western blot analysis showed that the expression of xCT was higher in CRC tissues than in normal tissues. **I** Relationship between xCT expression and clinicopathological characteristics in CRC patients from our collected CRC samples. **J** The prognosis of CRC patients with high expression of xCT was worse than that of CRC patients with low expression of xCT in GSE87211. **K** The ROC curve shows that xCT levels could predict the prognosis of CRC patients with great accuracy. **L** CRC patients with high expression of xCT had a poorer prognosis than those with low expression of xCT in our validation cohort. **M** The ROC curve confirmed the prognostic predictive accuracy in our validation cohort. **P < 0.01, ****P < 0.0001.

First, we conducted an overall study of the expression characteristics of xCT in a total of 24 types of tumors from TCGA database (Fig. 1A, B). We found that tumors in the intestine, including COAD and rectum adenocarcinoma (READ), exhibited higher expression of xCT than normal samples (Fig. 1C, D). We also confirmed the expression characteristics of xCT in CRC samples in GSE87211 and showed that CRC patients exhibited higher expression of xCT than normal subjects (Fig. 1E). Then, we also collected 49 CRC samples as an external validation cohort, and the IHC results were in accordance with this finding (Fig. 1F, G). We also found higher expression of xCT in CRC tissues than in normal samples through western blot analysis (Fig. 1H). In addition, poorer clinicopathological characteristics were observed in CRC patients with higher expression of xCT (Fig. 1I). CRC patients with high expression of xCT had a worse prognosis than those with low expression of xCT in GSE87211 (Fig. 1J), and area under the receiver operating characteristic (ROC) curve (AUC) was 0.825 (Fig. 1K). A consistent result was found in the validation cohort, where the AUC reached 0.89 (Fig. 1L, M). These findings reasonably indicate that xCT might play a positive role in the tumorigenesis of CRC and might be related to poor prognosis in CRC.

### xCT contributes to CRC cell proliferation and colony formation

**Fig. 2:**
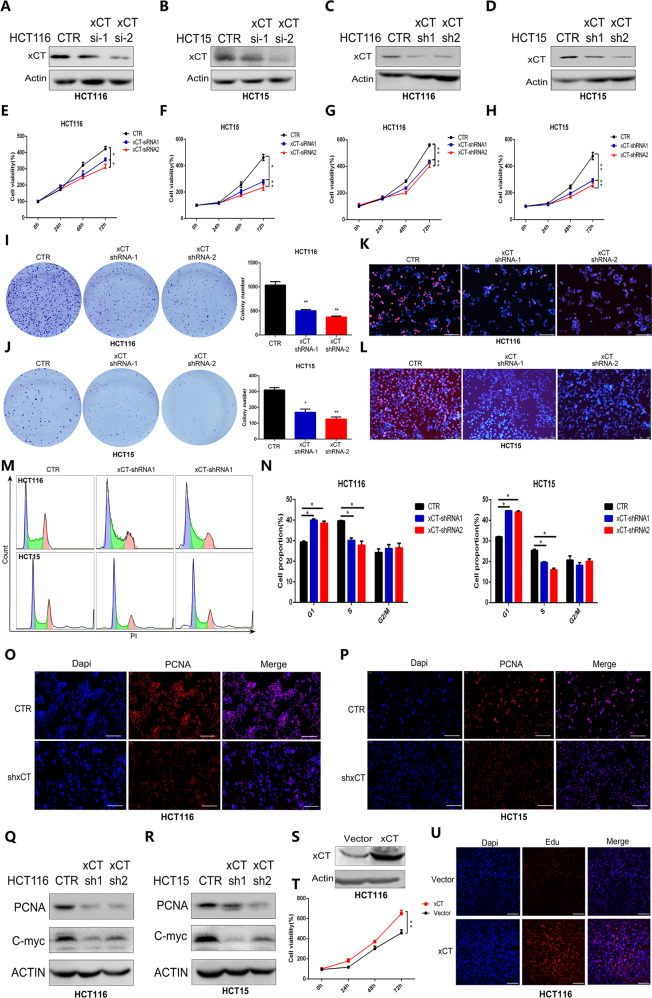
xCT contributes to proliferation and colony formation in CRC cells. **A**–**D** Western blot assay confirming the knockdown efficiency of xCT siRNA and xCT shRNA in CRC cells. **E**–**H** CCK-8 assay showed that xCT knockdown by both xCT siRNA and xCT shRNA significantly suppressed the proliferation of HCT116 and HCT15 cells. **I**, **J** Colony formation assays indicated that xCT inhibition markedly reduced HCT116 and HCT15 cell colony formation ability. **K**, **L** EdU assay confirmed that xCT inhibition attenuated HCT116 and HCT15 cell proliferation. **M**, **N** Cell cycle assay indicated that xCT inhibition hampered G1/S transition of HCT116 and HCT15 cells. **O**, **P** IF staining showed that xCT knockdown diminished the expression of PCNA in HCT116 and HCT15 cells. **Q**, **R** Western blot analysis revealed that xCT inhibition downregulated the expression of proliferation-related factors, including PCNA and C-myc, in HCT116 and HCT15 cells. **S** Western blot analysis indicated that xCT was overexpressed in HCT116 cells upon transfection. **T** CCK-8 assay showed that overexpression of xCT significantly promoted the proliferation of HCT116 cells. **U** EdU assay confirmed the promoting effect of xCT overexpression on the proliferation of HCT116 cells. *P < 0.05, **P < 0.01.

To explore the role of xCT in the proliferation of CRC, we knocked down the expression of xCT in HCT116 and HCT15 cells by transient siRNA transfection and lentiviral shRNA transfection. Western blotting showed that both methods effectively inhibited the expression of xCT in CRC cells (Fig. 2A–D). The CCK-8 assay indicated that xCT inhibtion significantly suppressed the proliferation of CRC cells (Fig. 2E–H). The colony formation assay showed that the colony formation ability of CRC cells with xCT inhibition was effectively reduced (Fig. 2I, J), and the finding of the EdU assay was consistent with this result (Fig. 2K, L). Cell cycle assay subsequently indicated that the xCT inhibition significantly increased the proportion of HCT116 and HCT15 cells in G1 phase and reduced the proportion of the cells in S phase (*P* < 0.05) (Fig. 2M, N). IF assays indicated that xCT inhibition significantly downregulated PCNA expression in CRC cells (Fig. 2O, P). Western blot analysis confirmed that the expression of PCNA and C-myc was significantly suppressed in CRC cells with xCT inhibition (Fig. 2Q, R). Western blot analysis indicated that xCT was overexpressed in HCT116 cells upon transfection (Fig. 2S). In contrast, CCK-8 and EdU assays confirmed that the overexpression of xCT promoted the proliferation of HCT116 cells (Fig. 2T, U). The findings above reveal that upregulated xCT expression effectively enhances the proliferation of CRC cells.

### xCT ablation suppresses the migration of CRC cells

**Fig. 3:**
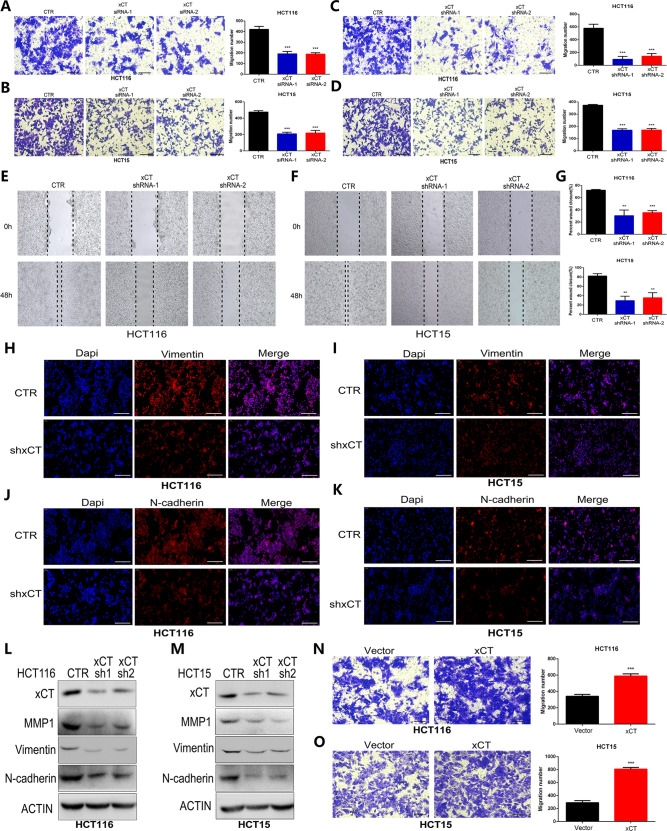
Upregulation of xCT in CRC cells promotes their migration and reverses the EMT process. **A**–**D** Transwell assays indicated that xCT knockdown with xCT siRNA and xCT shRNA significantly suppressed the migration of CRC cells. **E**–**G** The wound scratch assay and quantitative analysis showed that xCT knockdown inhibited the migration of CRC cells. **H**–**K** IF staining indicated that xCT inhibition reduced the expression of vimentin (**H**–**I**) and N-cadherin (**J**–**K**) in CRC cells. **L**, **M** Western blot analysis confirmed that xCT knockdown decreased the expression of MMP1 and vimentin in CRC cells. **N**, **O** Transwell assays showed that overexpression of xCT promoted the migration of CRC cells. **P < 0.01, ***P < 0.001.

The aggressiveness and metastatic capability of tumors are crucial factors affecting the treatment efficacy and prognosis; therefore, we next analyzed the role of xCT expression on the migration of HCT116 and HCT15 cell lines. Transwell assays showed that both xCT siRNA and xCT shRNA significantly suppressed the migration of CRC cells (Fig. 3A–D). The suppressive effect of xCT inhibition on the migration capability of CRC cells was also validated with a wound scratch assay (Fig. 3E–G). The expression characteristics of vimentin and N-cadherin play an important role in tumor metastasis and are commonly used to assess the migration ability of cells. IF assays showed that xCT inhibition significantly suppressed the expression of vimentin and N-cadherin in CRC cells (Fig. 3H–K). Western blot analysis also showed significant downregulation of MMP1 and vimentin expression in CRC cells transfected with xCT shRNA (Fig. 3L, M). Conversely, migration was significantly promoted in CRC cells overexpressing xCT (Fig. 3N, O). These findings confirm that xCT expression effectively increases the expression of migration- and metastasis-associated proteins in CRC cells.

### xCT inhibition restrains CRC cell stemness and decreases the expression of stemness-associated proteins in CRC cells

**Fig. 4:**
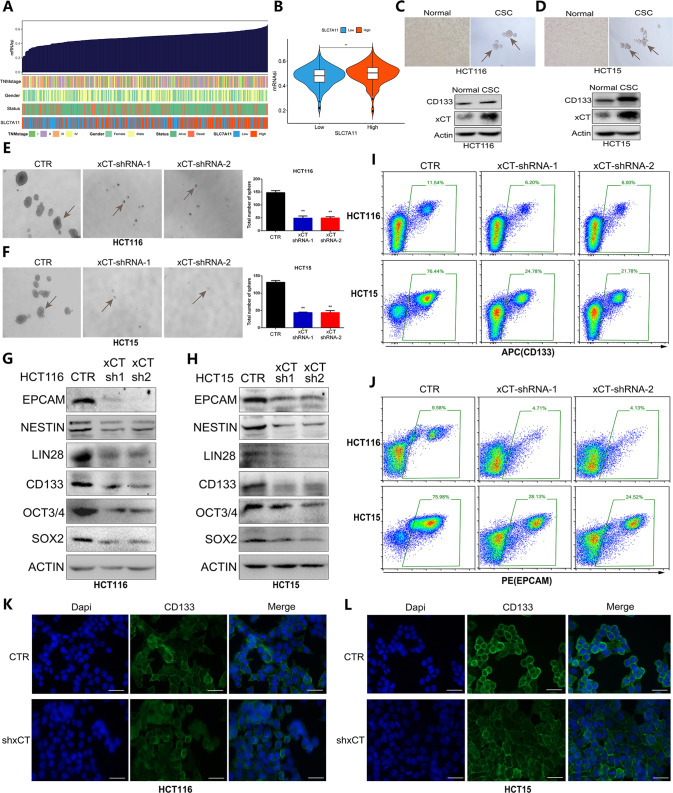
Effects of xCT knockdown on the stemness of HCT116 and HCT15 CRC cells. **A** An overview of the correlations between xCT expression, mRNAsi, and known clinical characteristics (TNM stage, sex, and survival status) in the TCGA-COAD cohort. **B** The association between the expression of xCT and mRNAsi in the TCGA-COAD cohort. **C**, **D** Western blot assay was applied to detect the expression of xCT in CRC cells and CRC stem cells. **E**, **F** Sphere formation assays showed that xCT inhibition suppressed the sphere-forming capability of CRC stem cells. **G**, **H** Western blot analysis showed that xCT knockdown reduced the expression level of stemness-related molecules in HCT116 cells and HCT15 cells. **I**, **J** Flow cytometry indicated that xCT inhibition significantly diminished the expression of EPCAM and CD133 in CRC cells. **K**, **L** IF staining showed that xCT knockdown suppressed the expression of CD133 in CRC cells. **P < 0.01.

To further confirm the role of xCT in CRC progression, the effect of xCT expression on cell stemness was also explored in CRC cells. First, we assessed the mRNAsi, which can be regarded as a quantification of CSCs, to explore the association of cell stemness with xCT expression and clinical features. Figure 4A, B show that the expression of xCT was positively correlated with the mRNAsi. Then, western blotting was used to detect the expression level of xCT in CRC cells and CRC stem cells (Fig. 4C, D). Sphere formation assays indicated that the sphere-forming capability of CRC stem cells was significantly suppressed by xCT inhibition (Fig. 4E, F). Western blotting also confirmed that xCT inhibition markedly decreased the expression level of stemness-related factors, such as EPCAM, CD133, NESTIN, and LIN28, in CRC cells (Fig. 4G, H). Flow cytometry further revealed a significant reduction in EPCAM and CD133 in CRC cells with xCT knockdown (Fig. 4I, J). IF staining also showed that xCT inhibition significantly reduced the expression of CD133 in CRC cells (Fig. 4K, L). The above results reasonably confirm that the expression of xCT is closely correlated with the stemness of CRC cells, suggesting that xCT plays a positive role in promoting the occurrence and development of CRC.

### Silencing of xCT suppresses the growth of CRC tumors and the lung metastatic capacity of CRC cells

**Fig. 5:**
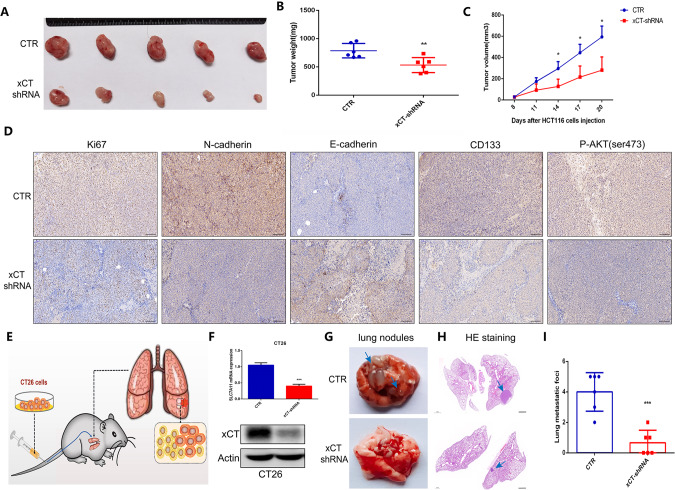
Silencing of xCT suppresses CRC growth and blocks spontaneous lung metastasis of CRC cells. **A** xCT knockdown inhibited the growth of tumors in CRC xenograft mouse models. Differences in tumor weight (**B**) and volume (**C**) in nude mouse CRC models subcutaneously injected with HCT116 cells with or without xCT inhibition. **D** IHC staining showed the changes in the expression of Ki67, N-cadherin, E-cadherin, CD133 and P-AKT in CRC tissues after xCT knockdown. **E** The process to establish a CRC lung metastasis mouse model. **F** qRT-PCR and western blotting assays confirmed the knockdown efficiency with xCT shRNA administration in CT26 cells. Macroscopic changes in the lungs (**G**) and pathological changes in the lung tissues (**H**) of mice after caudal vein injection of CT26 cells with or without xCT inhibition. **I** xCT knockdown significantly inhibited the metastatic capability of CRC. *P < 0.05, **P < 0.01, ***P < 0.001.

To further determine the role of xCT expression in tumor occurrence and progression in vivo, we constructed nude mouse CRC models by subcutaneously injecting HCT116 cells with or without xCT shRNA. Figure 5A indicates that xCT knockdown significantly suppressed the growth of tumors, as indicated by lighter tumor weights (Fig. 5B) and slower tumor volume growth (Fig. 5C) in nude mouse CRC models subcutaneously injected with HCT116 cells with xCT inhibition. IHC assays indicated xCT knockdown attenuated the expression of Ki67, N-cadherin, and CD133 (Fig. 5D). In addition, we further established a CRC lung metastasis mouse model via caudal vein injection of CT26 cells with or without xCT shRNA, and the operation process is shown in Fig. 5E. The knockdown efficiency of xCT shRNA in CT26 cells was confirmed by qRT-PCR and western blot assays (Fig. 5F). Macroscopic changes in the lungs (Fig. 5G) and pathological changes in lung tissues from mice (Fig. 5H, I) indicated that xCT inhibition weakened the metastatic capability of CRC. These findings suggest that xCT expression plays a positive role in tumor growth, migration and metastasis.

### E2F1 directly regulates the transcription of SLC7A11

**Fig. 6:**
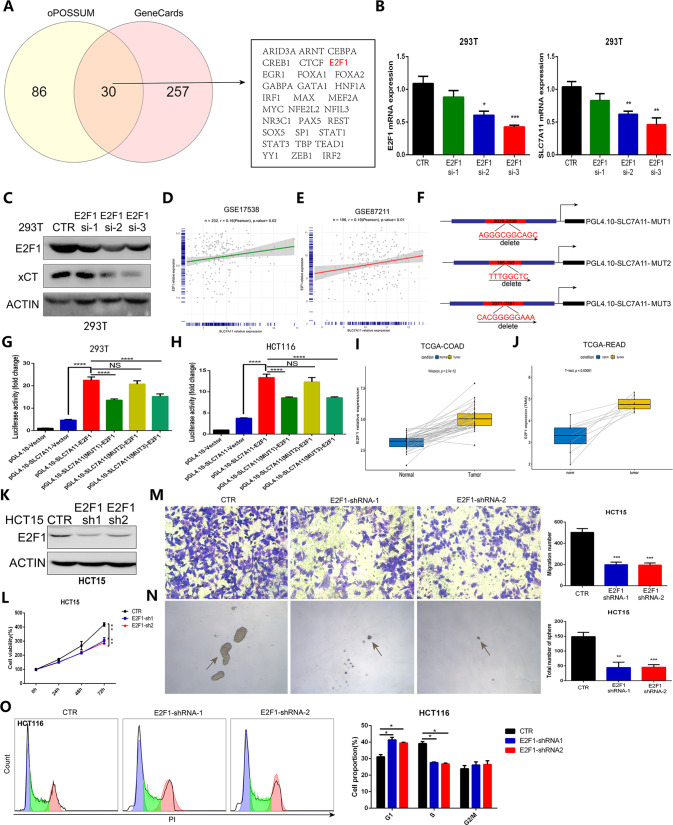
E2F1 was identified and confirmed as an upstream transcription factor of SLC7A11. **A** Prediction of potential upstream transcription factors of SLC7A11 in the oPOSSUM and GeneCards databases. **B** qRT-PCR showed that knockdown of E2F1 significantly suppressed the expression of SLC7A11. **C** Western blot analysis confirmed that E2F1 inhibition led to the downregulation of xCT expression in 293T cells. **D**, **E** The correlation between the expression of E2F1 and SLC7A11 in GSE17538 and GSE87211. **F** The potential binding sites of E2F1 to the SLC7A11 promoter region and binding site mutation strategy. **G**, **H** A luciferase assay was used to identify the binding sites of E2F1 to the SLC7A11 promoter region in 293T cells and HCT116 cells. **I**, **J** The expression of E2F1 was higher in CRC tissues than in normal samples from the TCGA-COAD cohort and TCGA-READ cohort. **K** Knockdown efficiency of E2F1 via lentiviral shRNA transfection was confirmed through western blot assay. **L** CCK-8 assay showed that E2F1 knockdown significantly inhibited the proliferation of HCT15 cells. **M** Transwell assays indicated that E2F1 inhibition effectively weakened the migration capability of HCT15 cells. **N** Sphere formation assays confirmed that E2F1 inhibition significantly suppressed the stemness of HCT15 cells. (O) Cell cycle assay showed E2F1 regulated cell cycle by inducing the G1/S transition. *P < 0.05, **P < 0.01, ***P < 0.001, ****P < 0.0001.

Then, we subsequently explored the potential upstream factor that mediates the expression of the SLC7A11 gene, which encodes xCT. We analyzed the potential upstream transcription factors of SLC7A11 in the oPOSSUM (http://opossum.cisreg.ca/oPOSSUM3/) and GeneCards databases (https://www.genecards.org/) (Fig. 6A), and the qRT-PCR and western blot results showed that the inhibition of E2F1 via siRNA transfection significantly suppressed SLC7A11 mRNA expression (Fig. 6B, C). Then, we explored the correlation between the expression of E2F1 and SLC7A11 in GSE17538 and GSE87211 and found that the expression of E2F1 showed a relatively positive correlation with that of SLC7A11 (Fig. 6D, E). Subsequently, we predicted three potential binding sites of E2F1 to the SLC7A11 promoter region in the JASPAR database (http://jaspar.genereg.net/), and the sequences of these potential binding sites are shown in Fig. 6F. We obtained the mutant E2F1(MUT1, MUT2, and MUT3) by deleting the sequence of the three binding sites separately. and then used a luciferase assay to identify the binding sites of E2F1 to the SLC7A11 promoter region. The results indicated that site 1 and site 3 may be the binding sites of E2F1 to the SLC7A11 promoter region (Fig. 6G, H). In addition, we assessed and confirmed that the expression of E2F1 in COAD and READ tissues was higher than that in normal samples in the TCGA database (Fig. 6I, J). To explore the role of E2F1 in the proliferation, migration, and stemness of CRC cells, we knocked down the expression of E2F1 via lentiviral shRNA transfection (Fig. 6K). The CCK-8 assay showed that E2F1 inhibition significantly suppressed the proliferation of HCT15 cells (Fig. 6L). The transwell assay also indicated that E2F1 knockdown effectively inhibited the migration of HCT15 cells (Fig. 6M), and the sphere formation assay results confirmed that the stemness of HCT15 cells with E2F1 knockdown was significantly inhibited compared with that of control HCT15 cells (Fig. 6N). Cell cycle assay showed E2F1 is a crucial factor enabling the G1-S transition of HCT116 cells (Fig. 6O). The findings above indicate that E2F1 is a potential upstream transcription factor of SLC7A11 and E2F1 also plays a role in promoting the progression of CRC.

### xCT activates the AKT/mTOR signaling pathway and enhances MELK expression in CRC cells

**Fig. 7:**
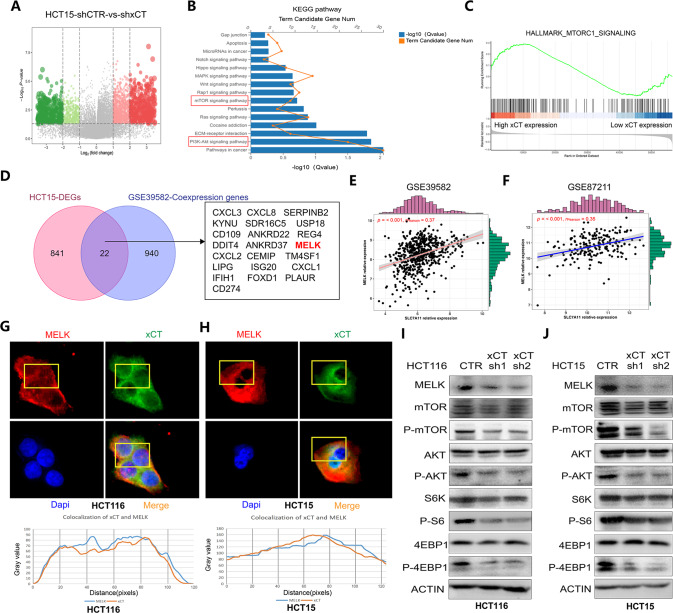
xCT activates the AKT/mTOR signaling pathway and enhances MELK expression in CRC cells. **A** DEGs in HCT15 cells with or without xCT shRNA administration. **B** KEGG analysis was performed on the DEGs. **C** GSEA showed that xCT was upregulated in samples with the activation of the hallmark_mTORC1_signaling pathway. **D** Identification of potential factors whose expression might correlate with that of xCT in CRC. **E**, **F** The correlation between the expression of MELK and xCT in GSE39582 and GSE87211. **G**, **H** IF assays confirmed that MELK and xCT were strongly colocalized in HCT116 and HCT15 cells. **I**, **J** Western blot analysis showed that xCT knockdown inhibited the expression level of MELK and restrained the activation of the AKT/mTOR signaling pathway in HCT116 and HCT15 cells.

To further explore the molecular mechanisms by which xCT expression regulates the tumorigenesis and progression of CRC, we analyzed DEGs in HCT15 cells with or without xCT knockdown (Fig. 7A). KEGG analysis showed that the DEGs were enriched in signaling pathways such as the “PI3K-AKT signaling pathway” and “mTOR signaling pathway” (Fig. 7B). The gene set enrichment analysis (GSEA) results also showed that samples with high expression of xCT showed high enrichment of genes in the hallmark_mTORC1_signaling pathway (Fig. 7C). In addition, through the integrated analysis of the DEGs from HCT15 cells and coexpressed genes from the GSE39582 dataset, we identified potential factors whose expression might be related to that of xCT (Fig. 7D). Interestingly, among these factors, MELK has also been revealed in previous studies to activate the mTORC1 and mTORC2/AKT signaling pathways and promote cancer progression when expressed at high levels [5]. Based on this finding, we analyzed the correlation between the expression of MELK and xCT in GSE39582 and GSE87211, and the results revealed a positive correlation (Fig. 7E, F). Then, the results of IF assays further confirmed that MELK and xCT were strongly colocalized in CRC cells (Fig. 7G, H). Western blot assays showed that xCT knockdown significantly decreased the expression of MELK and the phosphorylation levels of AKT, mTOR, and important substrates of mTORC1 (S6K and 4E-BP1) in CRC cells, while there were no significant changes in the total expression of AKT, mTOR, S6K, and 4E-BP1 (Fig. 7I, J). These results indicate that xCT knockdown inhibits MELK expression and AKT/mTOR signaling pathway, thereby affecting the tumorigenesis and progression of CRC.

### MELK is required for xCT-mediated CRC tumorigenesis and AKT/mTOR signaling

**Fig. 8:**
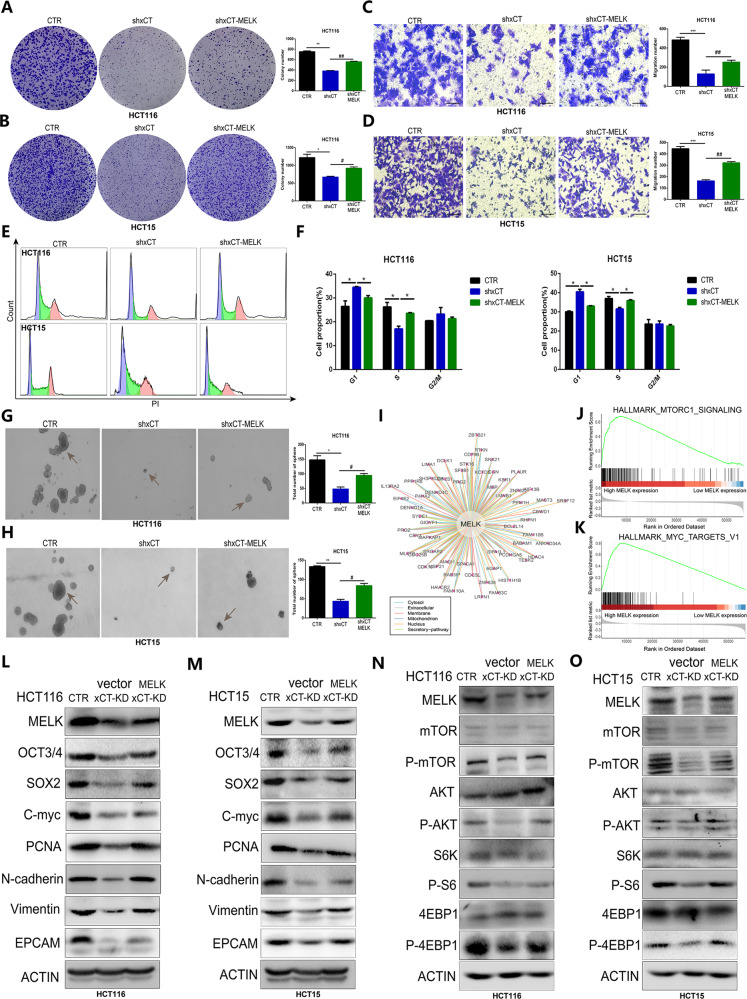
MELK is required for xCT-mediated CRC tumorigenesis and AKT/mTOR signaling. **A**, **B** The colony formation assay showed that the suppressive effect of xCT inhibition on the proliferation of CRC cells was reversed by MELK upregulation. **C**, **D** Transwell assays indicated that MELK overexpression effectively recovered the migration capability inhibited by xCT knockdown in CRC cells. **E**, **F** Cell cycle assay confirmed that MELK rescued the suppression of xCT inhibition on G1/S transition. **G**, **H** The sphere formation assay indicated that upregulated MELK rescued the suppressive effect of xCT inhibition on the sphere-forming capability of CRC cells. **I** The interaction of MELK with different factors expressed in various organelles. **J**, **K** GSEA indicated that upregulated expression of MELK was closely related to the activation of signaling pathways, including hallmark_mTORC1_signaling and hallmark_MYC_targets_V1. **L**, **M** Western blot analysis confirmed that upregulated MELK rescued the suppression of the expression of tumorigenesis- and stemness-related factors in CRC cells with xCT knockdown. **N**, **O** The suppressive effect of xCT knockdown on the AKT/mTOR signaling pathway was rescued by MELK overexpression. *P < 0.05, **P < 0.01, ***P < 0.001, ^#^ *P* < 0.05, ^##^ *P* < 0.01.

To further confirm how MELK is involved in the regulation of xCT-mediated CRC occurrence and progression, we overexpressed MELK in xCT-knockdown CRC cells, including HCT116 and HCT15 cells. A colony formation assay showed that the upregulation of MELK significantly rescued the antiproliferative effect of xCT inhibition on CRC cells (Fig. 8A, B). The inhibitory effect of xCT knockdown on CRC cell migration was also effectively reversed by augmentation of MELK (Fig. 8C, D). Then we subsequently determined MELK overexpression significantly rescued the suppression of xCT knockdown on G1-S transition (Fig. 8E, F). The xCT-mediated decrease in the stemness of CRC cells was also effectively rescued by MELK overexpression in HCT116 cells and HCT15 cells (Fig. 8G, H). Then, we explored the interaction of MELK with different factors expressed in various organelles (Fig. 8I). The GSEA results indicated that MELK upregulation was closely related to the activation of signaling pathways, including hallmark_mTORC1_signaling and hallmark_MYC_targets_V1 (Fig. 8J, K). In addition, western blot analysis showed that the expression of tumorigenesis- and stemness-related factors, such as PCNA, N-cadherin, and EPCAM, was rescued by MELK overexpression in xCT knockdown CRC cells (Fig. 8L, M). Moreover, MELK overexpression effectively reversed the xCT knockdown-induced suppression of AKT/mTOR signaling pathway activation (Fig. 8N, O). These findings reveal that MELK is required for xCT-mediated CRC tumorigenesis and AKT/mTOR signaling. And the overall mechanism by which E2F1/xCT promotes CRC stemness and malignancy was exhibited in Fig. S1.

### Treatment with the xCT inhibitor erastin inhibited the tumorigenesis and stemness of CRC

Erastin is a specific small-molecule inhibitor of xCT [19]. In this study, we treated CRC cells with different concentrations of erastin to further validate the effect of xCT on the tumorigenesis and progression of CRC. First, we measured the half-maximal inhibitory concentration (IC50) of erastin to ensure toxicity to CRC cells, and the results are shown in Fig. S2A, B. The next experiments were based on the IC50 of erastin in CRC cells. Colony formation assays indicated that the inhibitory effect of erastin on the proliferation of CRC cells was positively correlated with its treatment concentration (0–10 µM) (Fig. S2C, D). Transwell assays also revealed that erastin treatment significantly inhibited the migration of CRC cells (Fig. S2E, F). Further cell cycle assay indicated erastin treatment mainly played a inhibitory effect on G1-S transition of CRC cells (Fig. S2G, H). The inhibitory effect of erastin on the stemness of CRC cells also presented a positive correlation with the treatment concentration (0–10 µM) (Fig. S2I, J). We further assessed the antitumor effect of erastin in vivo, and it was confirmed that erastin treatment significantly inhibited the growth of tumors (Fig. S2K). HE staining indicated erastin treatment barely leads to organs toxicity in vivo (Fig. S3). Figure S2L–S2N show erastin treament also barely affected body weight, but effectively inhibited tumor volume growth and tumor weights in CRC. The changes in the expression of tumorigenesis- and stemness-related factors in tumor tissues with or without erastin treatment are shown in Fig. S2O. These results suggest xCT inhibitor erastin may be a potential targeted drug for controlling tumor progression.

## Discussion

As one of the major malignant cancers in the world, CRC is a serious threat to human health and presents a great public health burden to society [20]. As a highly molecular heterogeneous disease, the underlying molecular mechanism of CRC is still poorly understood [21]. Exploring the changes in special molecular characteristics in the tumorigenic process of CRC may be beneficial and ultimately may provide patients with more effective and personalized targeted therapy options and improve the prognosis of patients. In this study, we performed bioinformatics analysis and conducted IHC and Western blot analyses to validate the expression characteristic of xCT in CRC and explore its correlation with the prognosis of CRC. The expression of xCT in the TCGA database was not associated with the prognosis of CRC, but we confirmed that the expression level of xCT was significantly upregulated in CRC tissues compared with normal tissues we collected in clinic and high xCT expression was closely related to poor clinicopathological characteristics and a poor prognosis in CRC patients. And consistent results were also validated in the GEO database that high expression of xCT is strongly associated with poor prognosis in CRC patients. We hypothesized that this difference in results may be due to the different ethnicities of the study population, with the TCGA cohort predominantly American and the Cohort we validated for the Asian population. And it is worth mentioning that whether in the TCGA cohort or other databases, xCT is highly expressed in CRC tissues compared to normal tissues, and combined with studies published previously that xCT play a role as an oncogenic gene in tumor development, which is also consistent with our study result. Subsequent experiments validated that the knockdown of xCT effectively suppressed the proliferation and migration of CRC cells in vitro and limited the tumorigenicity and metastatic capability of CRC in vivo. In addition, the administration of erastin, a specific inhibitor of erastin, was shown to significantly slow the development of CRC. These findings indicate that xCT may serve as an effective potential molecular target to control the tumorigenesis of CRC and that erastin may be a potential drug option to inhibit the progression of CRC.

CSCs are defined as a subset of cancer cells that have the stem cell-like characteristics [22], and contrary to the physiological effects of normal stem cells, the enhanced self-renewal cloning, growth, metastasis, recurrence, and reproliferation capabilities of CSCs cannot maintain the dynamic balance of tissues but instead facilitate cancer progression [23, 24]. Importantly, the existence of CSCs has been confirmed in CRC tissues, and these cells have vital positive effects on the tumorigenicity, metastasis, and recurrence of CRC [25]. Our study revealed that xCT knockdown effectively suppressed the sphere-forming capability of CRC cells, and the expression of stemness-related factors such as CD133 and EPCAM was significantly downregulated with xCT knockdown in both CRC cells and tumor tissues, indicating that xCT knockdown plays a crucial role in suppressing the stemness of CRC cells. Based on these results, we can reasonably speculate that the expression of xCT is closely related to the stemness of CRC CSCs and that targeting the expression of xCT is an effective method for interfering with the stemness of CSCs.

When exploring the potential upstream transcription factors involved in regulating xCT expression, we used two online databases, oPOSSUM and GeneCards, for predictive analysis. Based on the predictive result, we first excluded the genes such as STAT3、NFE2L2 which have been validated as the upstream transcription factors of SLC7A11 in previous studies. Then we chose the “star factors” including SP1、YY1、ZEB1、E2F1、IRF1 which have not been reported the association with SLC7A11 but is common to be analyzed in previous researches to validate their effect on SLC7A11 expression. And eventually we confirmed that xCT may be targeted and directly regulated by the transcription factor E2F1, a necessary factor enabling the G1-S transition during the cell cycle and plays a pivotal role in mediating multiple cancer hallmark capabilities including survival, apoptosis, metabolism, and metastasis [26, 27]. Increased evidences have determined that aberrant activation of E2F1 is closely associated with a poor clinical outcome in various human cancers [28, 29], and it was shown that E2F1 played a positive role in colon cancer metastasis and drug resistance [30]. We validated that E2F1 directly regulates the transcription of SLC7A11 by qRT-PCR and Western blot assays and luciferase assays, and the potential binding sites of E2F1 and the SLC7A11 promoter region were determined in subsequent experiments. In addition, the knockdown of E2F1 was revealed to play a significant role in inhibiting the proliferation, migration, and stemness of CRC cells, indicating that E2F1 may also be a potential therapeutic target for CRC treatment.

In the subsequent exploration of the molecular mechanism of xCT affecting the tumorigenesis and progression of CRC, we found that the PI3K/Akt/mTOR axis may be a potential pivotal regulatory pathway, and it was further determined in our study that xCT played a crucial role in regulating the activity of Akt/mTOR signaling in CRC cells. We confirmed that xCT knockdown significantly reduced the phosphorylation levels of AKT, mTOR, and important substrates of mTORC1 (S6K and 4E-BP1), indicating that xCT may control the occurrence and development of tumors by interfering with the activation of Akt/mTOR signaling. To further detail the molecular mechanism by which xCT regulates the occurrence and progression of CRC, we explored and identified MELK as an essential factor that interacts with xCT and the Akt/mTOR signaling pathway. In our study, we determined that MELK and xCT were strongly colocalized in CRC cells using IF staining and that the expression of MELK was regulated by xCT. In recent years, many studies have shown that MELK is upregulated in multiple types of human tumors including lung, breast, and gastric cancers [31, 32], the effect of RNAi-mediated MELK knockout has confirmed that MELK expression is essential for the proliferation and invasion of cancer cells [33]. Interestingly, when analyzing the role of the Akt/mTOR signaling pathway in the tumorigenesis and stemness of cancer, we found previous studies indicating that high MELK expression can activate the mTORC1 and mTORC2/AKT signaling pathways and promote the development of endometrial cancer (EC) [5]. In our study, MELK overexpression effectively attenuated xCT knockdown-induced suppression of tumor proliferation, migration, and stemness, and Akt/mTOR signaling activity was also significantly restored, indicating that xCT may control the tumorigenesis and progression of CRC via the MELK/Akt/mTOR signaling pathway.

In summary, this study confirmed that xCT exerts an oncogenic effect in CRC by controlling tumorigenesis, metastasis, and cell stemness. E2F1 directly regulates the transcription of SLC7A11 and promotes the proliferation, migration, and stemness of CRC cells. In addition, xCT activates the AKT/mTOR pathway in CRC cells. MELK and xCT are strongly colocalized in CRC cells, and the expression of MELK is regulated by xCT. Interestingly, MELK is required for xCT-mediated CRC tumorigenesis and AKT/mTOR signaling pathway activation. Erastin, which was confirmed to effectively inhibit the tumorigenesis and cell stemness of CRC, may serve as a potential therapeutic drug in CRC treatment. Our findings may provide new options for therapeutic targets and drugs for the treatment of CRC.

## Supplementary information

Supplementary material Western Blots

Supplementary materials

FigureS1

FigureS2

FigureS3

supporting information

Revised Manuscript- Marked Up

checklist

## Data Availability

The data used to support the findings of this study are available from the corresponding author upon request.
