# Supplementary material for: xCT contributes to colorectal cancer tumorigenesis through upregulation of the MELK oncogene and activation of the AKT/mTOR cascade
Source: Cell Death Dis. 2022 Apr 19;13(4):373. doi: 10.1038/s41419-022-04827-4 (PMC9019093; doi:10.1038/s41419-022-04827-4)
Supplement: Supplementary file 1 — Supplementary material Western Blots [file 41419_2022_4827_MOESM1_ESM.pdf]

Supplementary Original Western Blots

Figure 1

1H

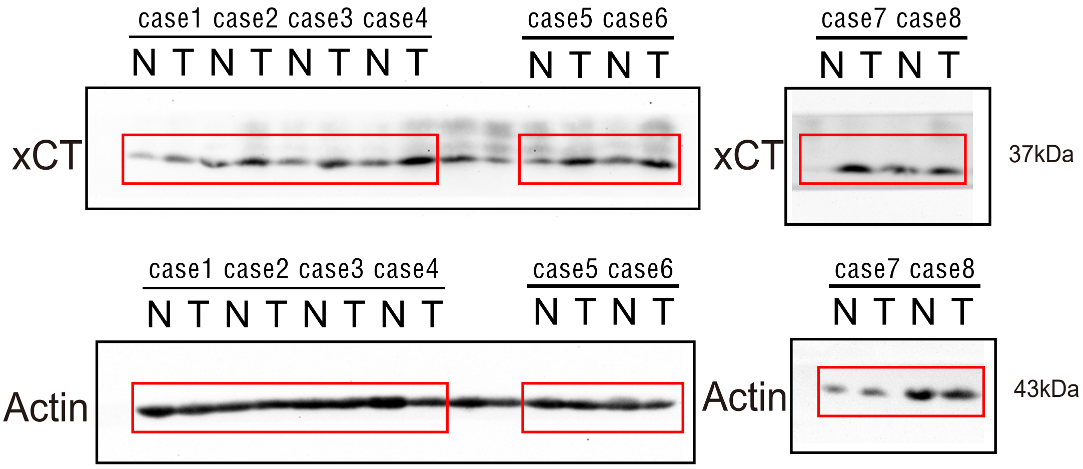

Figure 2

2A

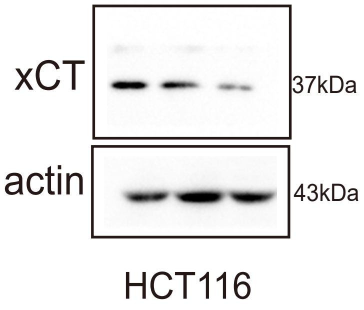

2B

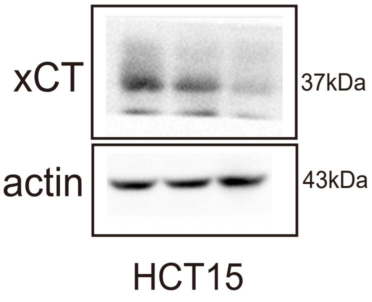

2C

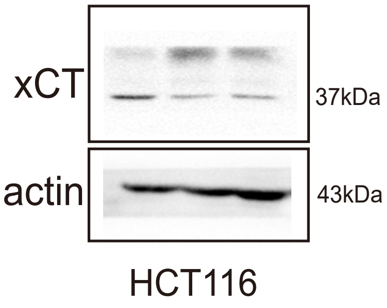

2D

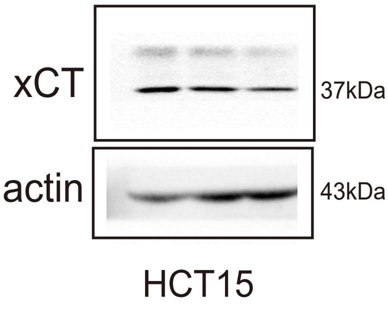

2Q

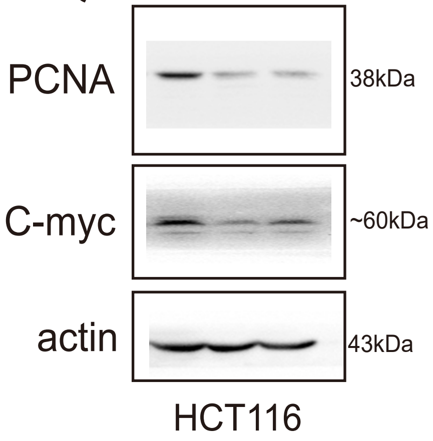

2R

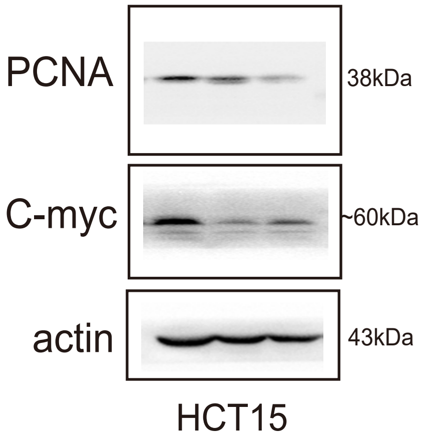

2S

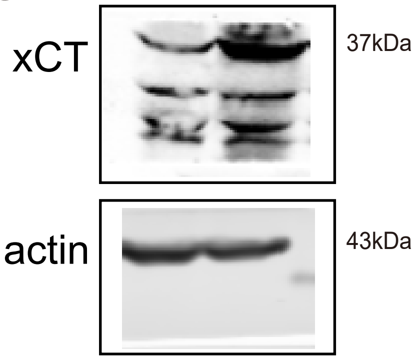

Figure 3

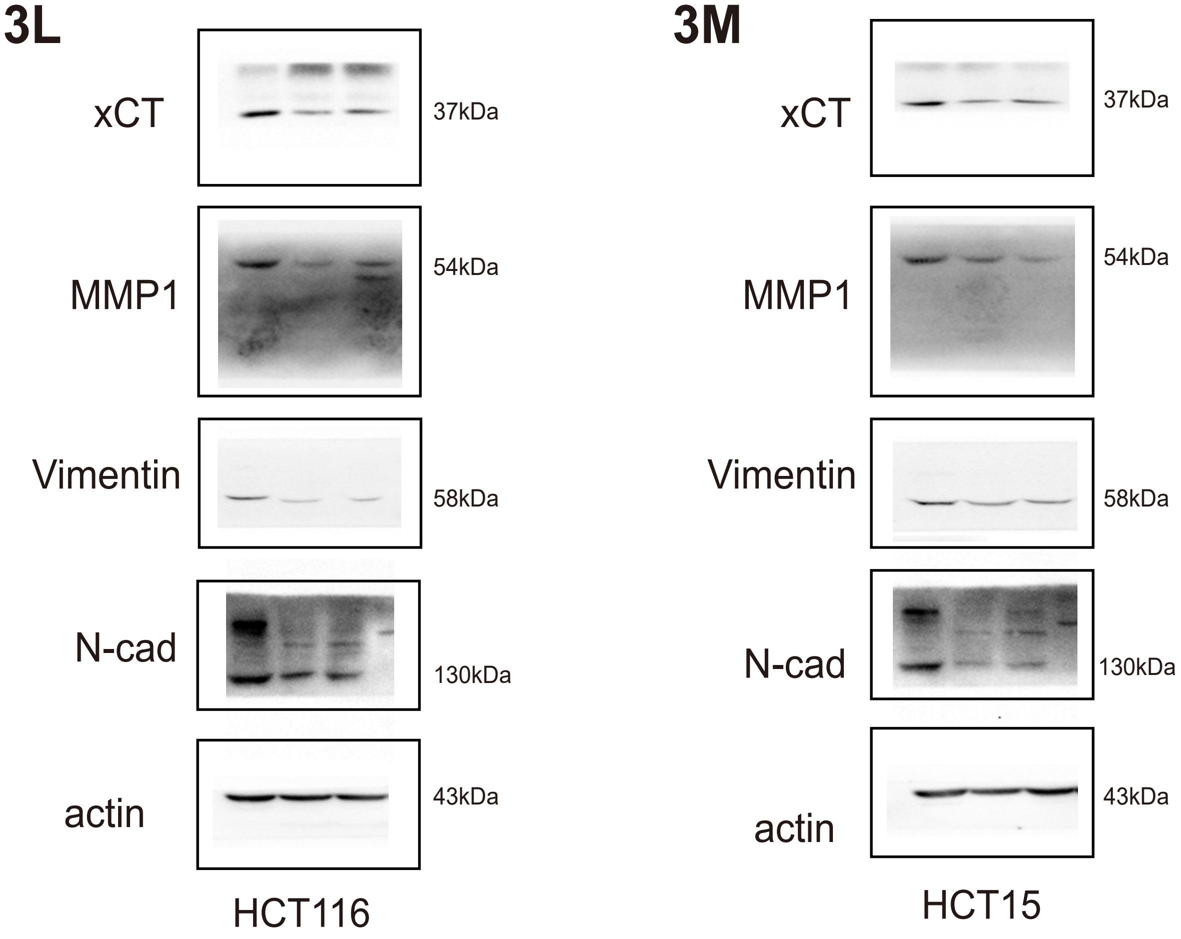
3L
xCT
37kDa
MMP1
54kDa
Vimentin
58kDa
N-cad
130kDa
actin
43kDa
HCT116
3M
xCT
37kDa
MMP1
54kDa
Vimentin
58kDa
N-cad
130kDa
actin
43kDa
HCT15

Figure 4

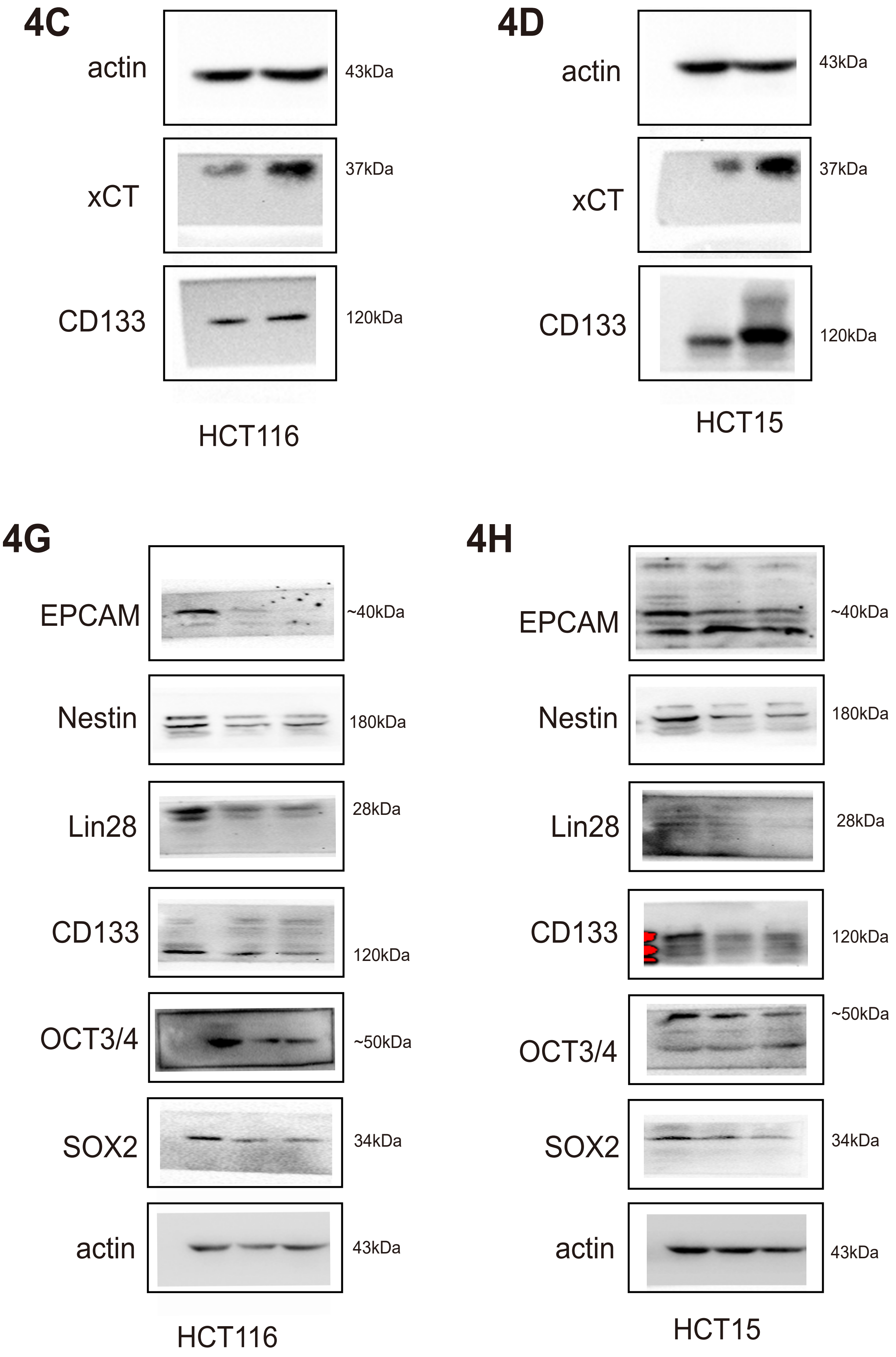
4C
actin
43kDa
xCT
37kDa
CD133
120kDa
HCT116
4D
actin
43kDa
xCT
37kDa
CD133
120kDa
HCT15
4G
EPCAM
~40kDa
Nestin
180kDa
Lin28
28kDa
CD133
120kDa
OCT3/4
~50kDa
SOX2
34kDa
actin
43kDa
HCT116
4H
EPCAM
~40kDa
Nestin
180kDa
Lin28
28kDa
CD133
120kDa
OCT3/4
~50kDa
SOX2
34kDa
actin
43kDa
HCT15

Figure 5

5F

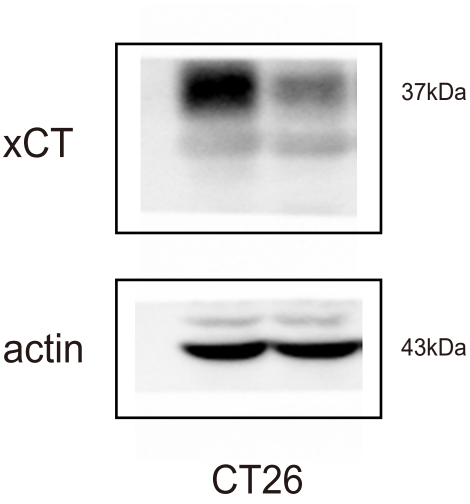

Figure 6

6C

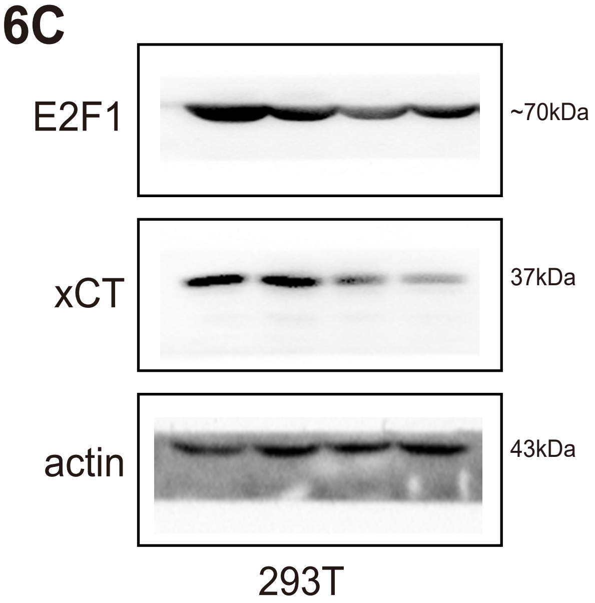

6K

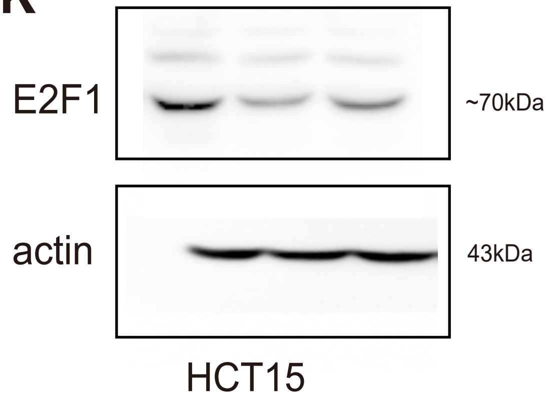

Figure 7

7I 7J

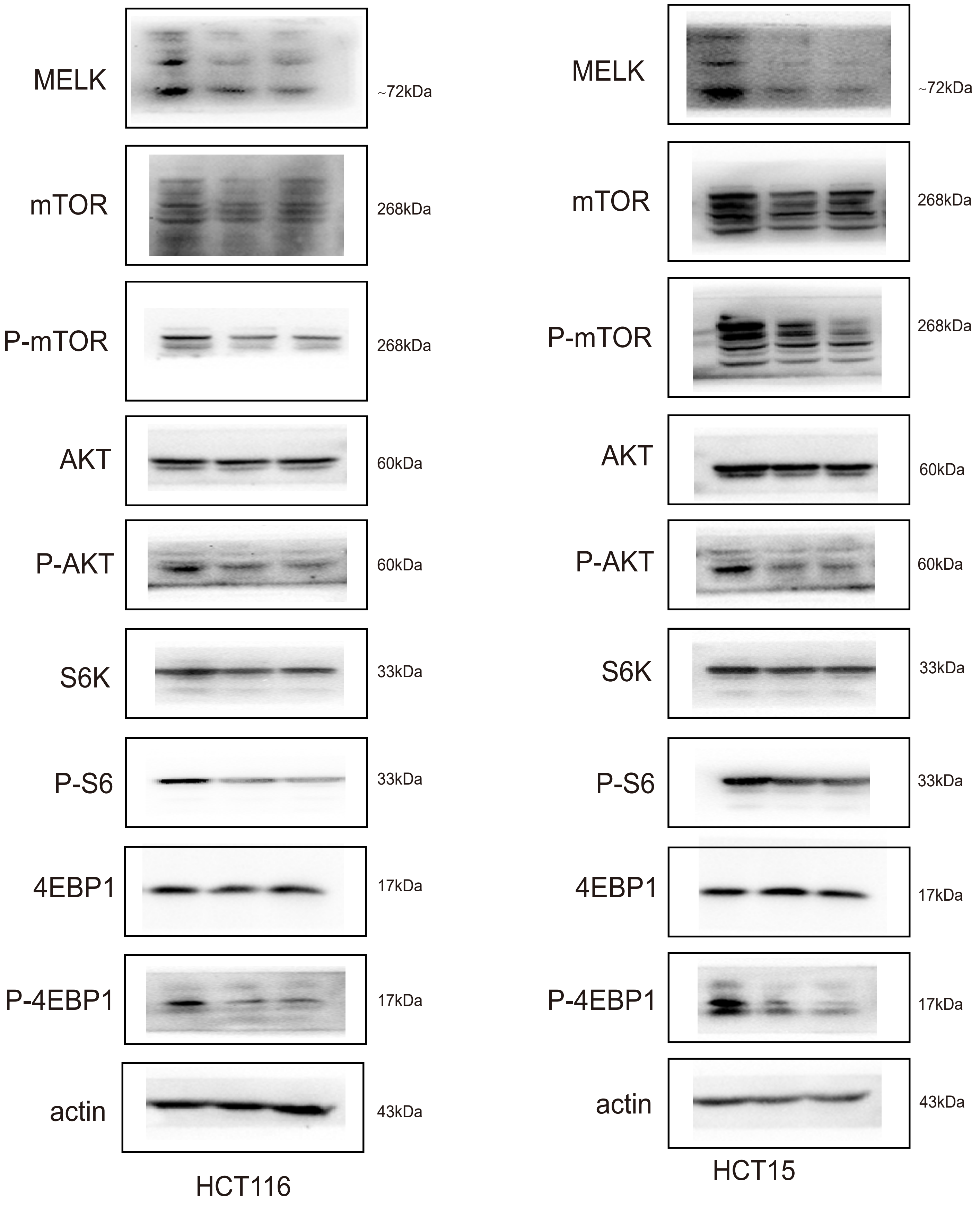

Figure 8

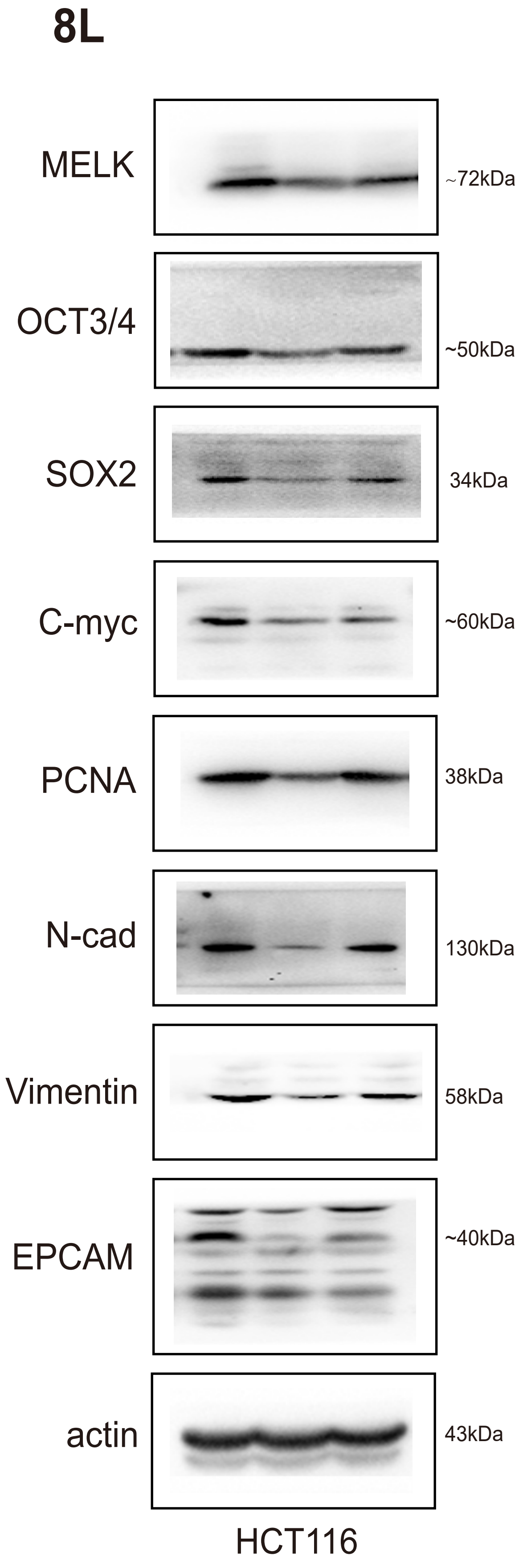
8L
MELK
~72kDa
OCT3/4
~50kDa
SOX2
34kDa
C-myc
~60kDa
PCNA
38kDa
N-cad
130kDa
Vimentin
58kDa
EPCAM
~40kDa
actin
43kDa
HCT116

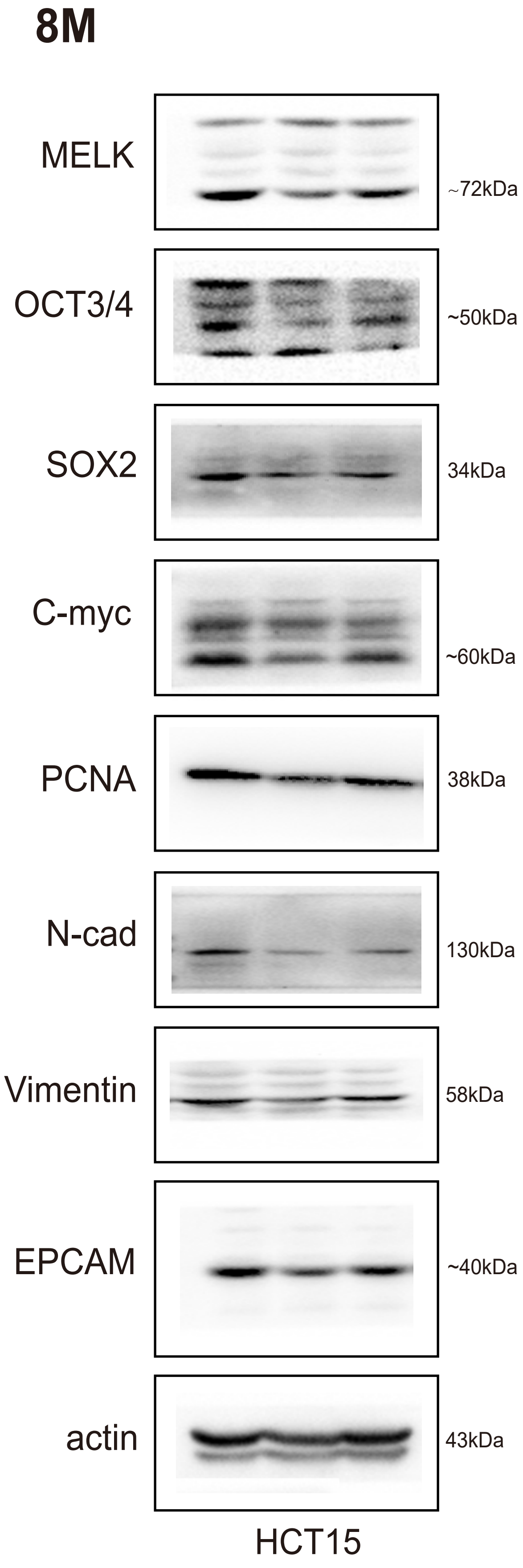
8M
MELK
~72kDa
OCT3/4
~50kDa
SOX2
34kDa
C-myc
~60kDa
PCNA
38kDa
N-cad
130kDa
Vimentin
58kDa
EPCAM
~40kDa
actin
43kDa
HCT15

8N

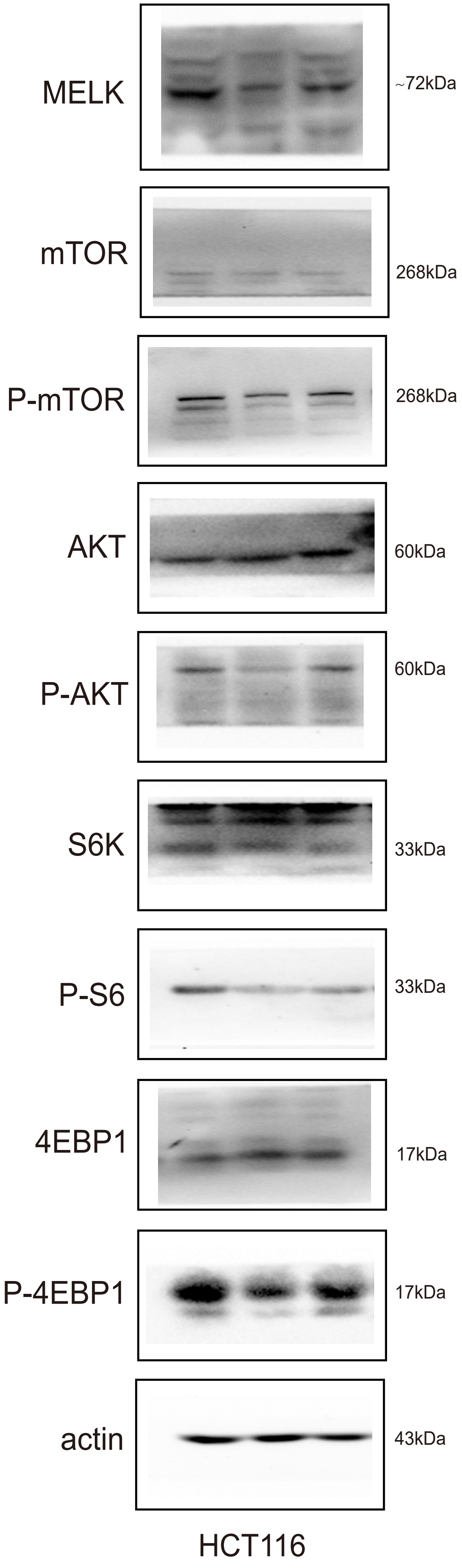
MELK
~72kDa
mTOR
268kDa
P-mTOR
268kDa
AKT
60kDa
P-AKT
60kDa
S6K
33kDa
P-S6
33kDa
4EBP1
17kDa
P-4EBP1
17kDa
actin
43kDa
HCT116

8O

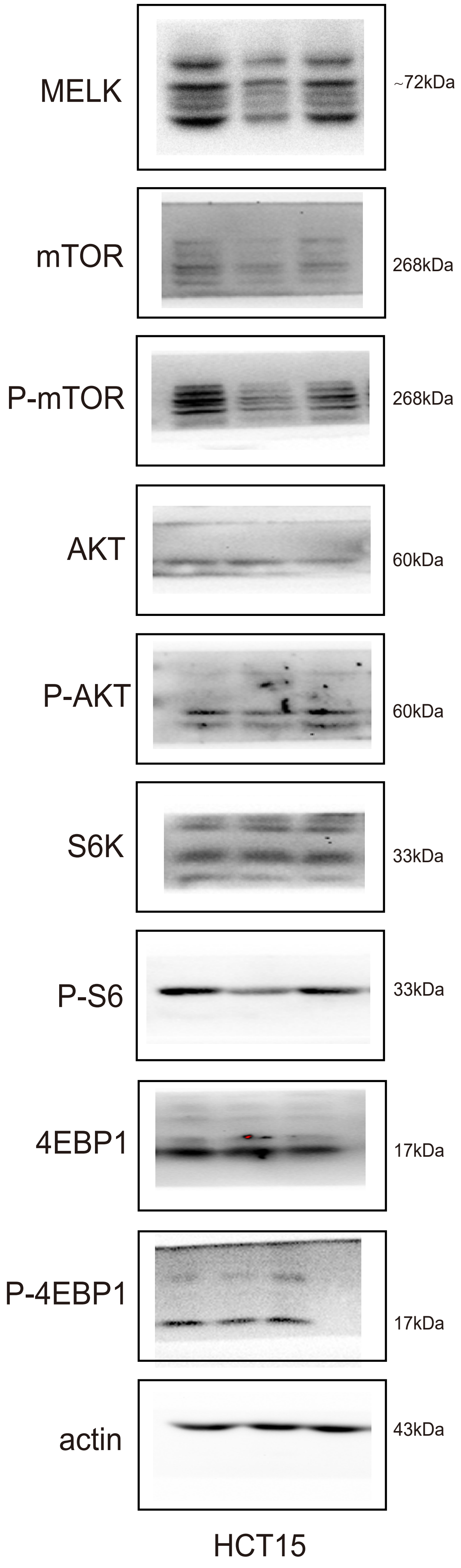
MELK
~72kDa
mTOR
268kDa
P-mTOR
268kDa
AKT
60kDa
P-AKT
60kDa
S6K
33kDa
P-S6
33kDa
4EBP1
17kDa
P-4EBP1
17kDa
actin
43kDa
HCT15
