# Supplementary material for: xCT contributes to colorectal cancer tumorigenesis through upregulation of the MELK oncogene and activation of the AKT/mTOR cascade
Source: Cell Death Dis. 2022 Apr 19;13(4):373. doi: 10.1038/s41419-022-04827-4 (PMC9019093; doi:10.1038/s41419-022-04827-4)

**Supplementary materials**

**Figure legends**

**Figure S1. Schematic of the mechanism by which E2F1/xCT promotes CRC stemness and malignancy.**

**Figure S2**. **Erastin treatment inhibits the tumorigenesis and stemness of CRC both in vitro and in vivo.** (**A-B**) Confirmation of the IC50 of erastin in HCT116 and HCT15 cells. (**C-D**) Colony formation assays showed that erastin treatment inhibited the proliferation of HCT116 and HCT15 cells. (**E-F**) Transwell assays showed the suppressive effect of erastin (20 µM) on the migration of HCT116 and HCT15 cells. (**G-H**) Cell cycle assay indicated that erastin treatment hampered G1/S transition of HCT116 and HCT15 cells. **(I-J)** Sphere formation assays confirmed that the stemness of HCT116 and HCT15 cells was significantly inhibited with erastin administration. (**K**) Erastin treatment inhibited tumor growth in CRC xenograft mouse models. (**L**) The body weight of the erastin treatment group was barely different from that of the control (saline) treatment group. **(M)** The tumor growth rate in the erastin treatment group was lower than that in the control treatment group. (**N**) The tumor weight in the erastin treatment group was lighter than that in the control treatment group. (**O**) IHC staining revealed that erastin treatment suppressed the expression of tumorigenesis- and stemness-related factors in CRC tissues. * P < 0.05, ** P < 0.01, *** P < 0.001.

**Figure S3. Erastin treatment barely lead to organs toxicity in vivo.** (**A**) HE staining indicated pathological changes of tumor tissue with or without erastin treatment. (**B-F**) HE staining implied that pathological features of mice organs including heart, liver, spleen, lung, kidney with or without erastin administration.

**Figure S1.**


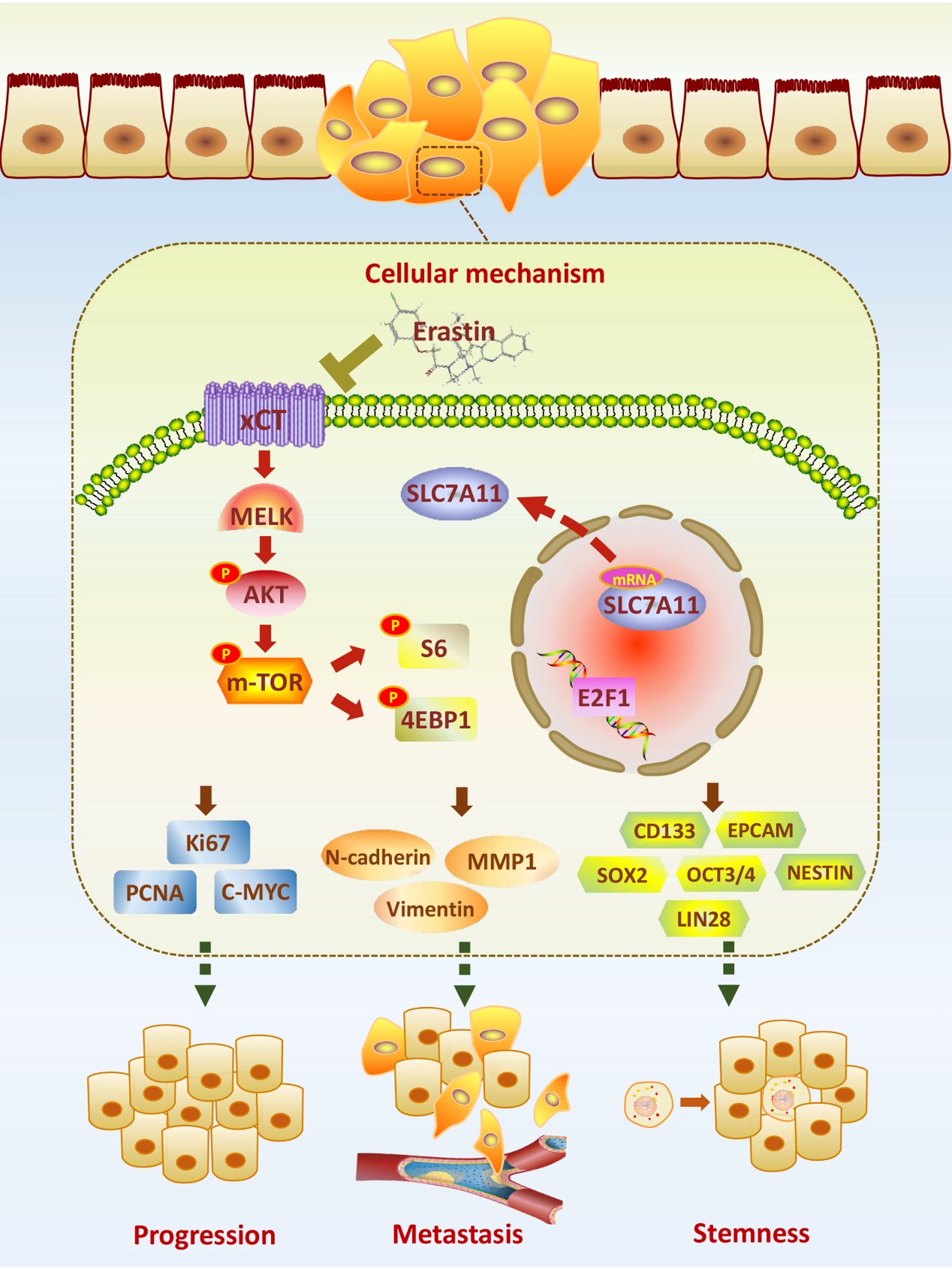


**Figure S2.**


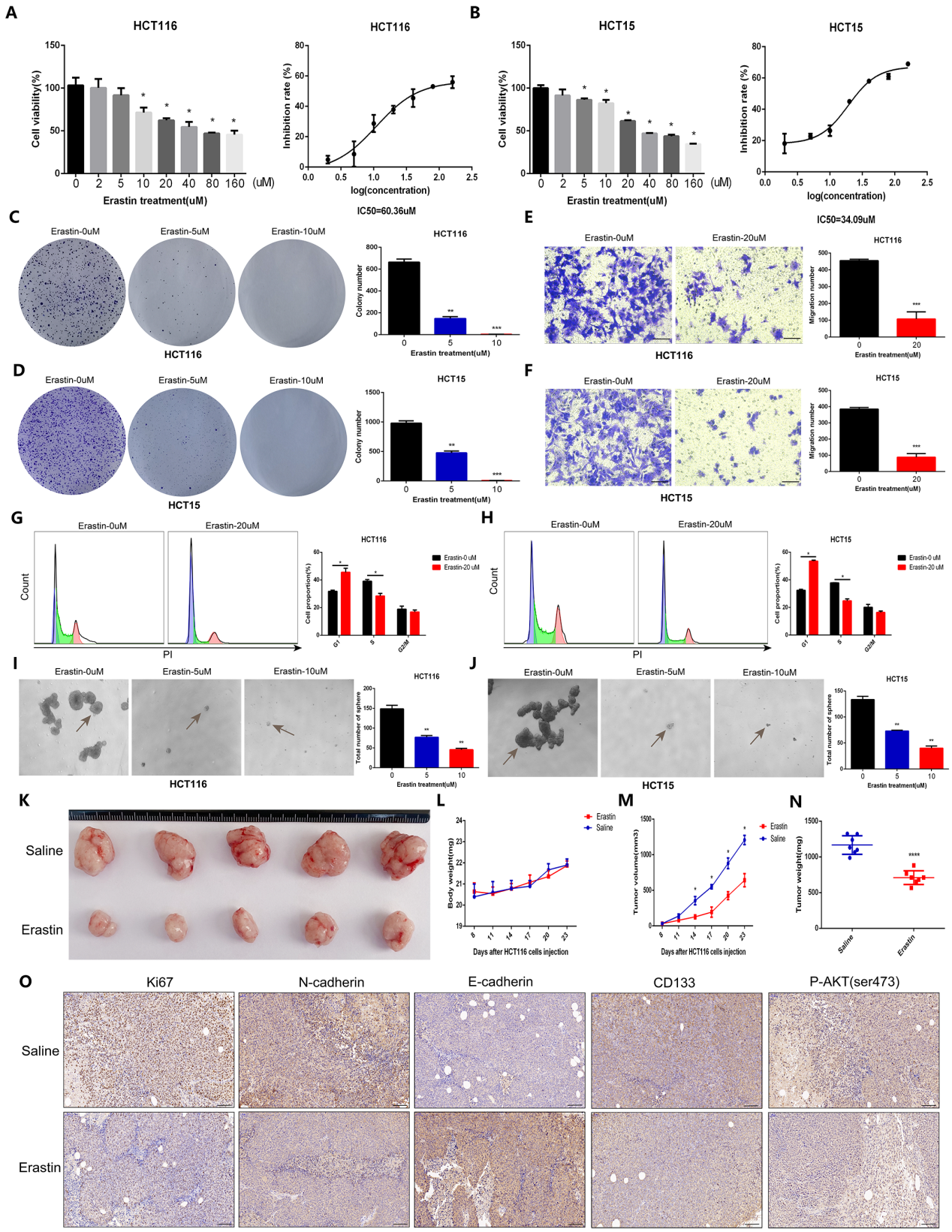


**Figure S3.**


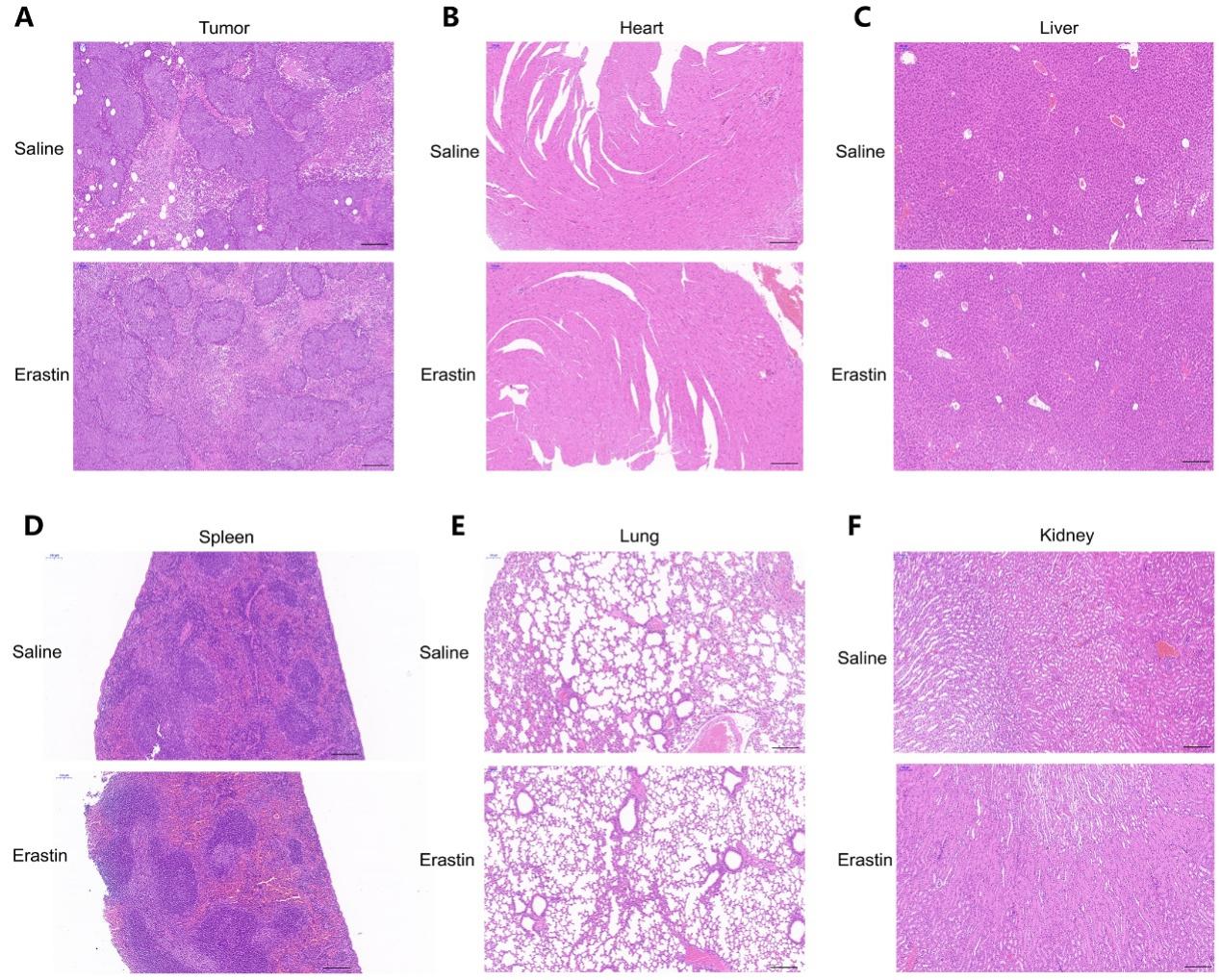

Supplement: Supplementary file 2 — Supplementary materials [file 41419_2022_4827_MOESM2_ESM.docx]
