# Supplementary figures and images for: xCT contributes to colorectal cancer tumorigenesis through upregulation of the MELK oncogene and activation of the AKT/mTOR cascade
Source: Cell Death Dis. 2022 Apr 19;13(4):373. doi: 10.1038/s41419-022-04827-4 (PMC9019093; doi:10.1038/s41419-022-04827-4)

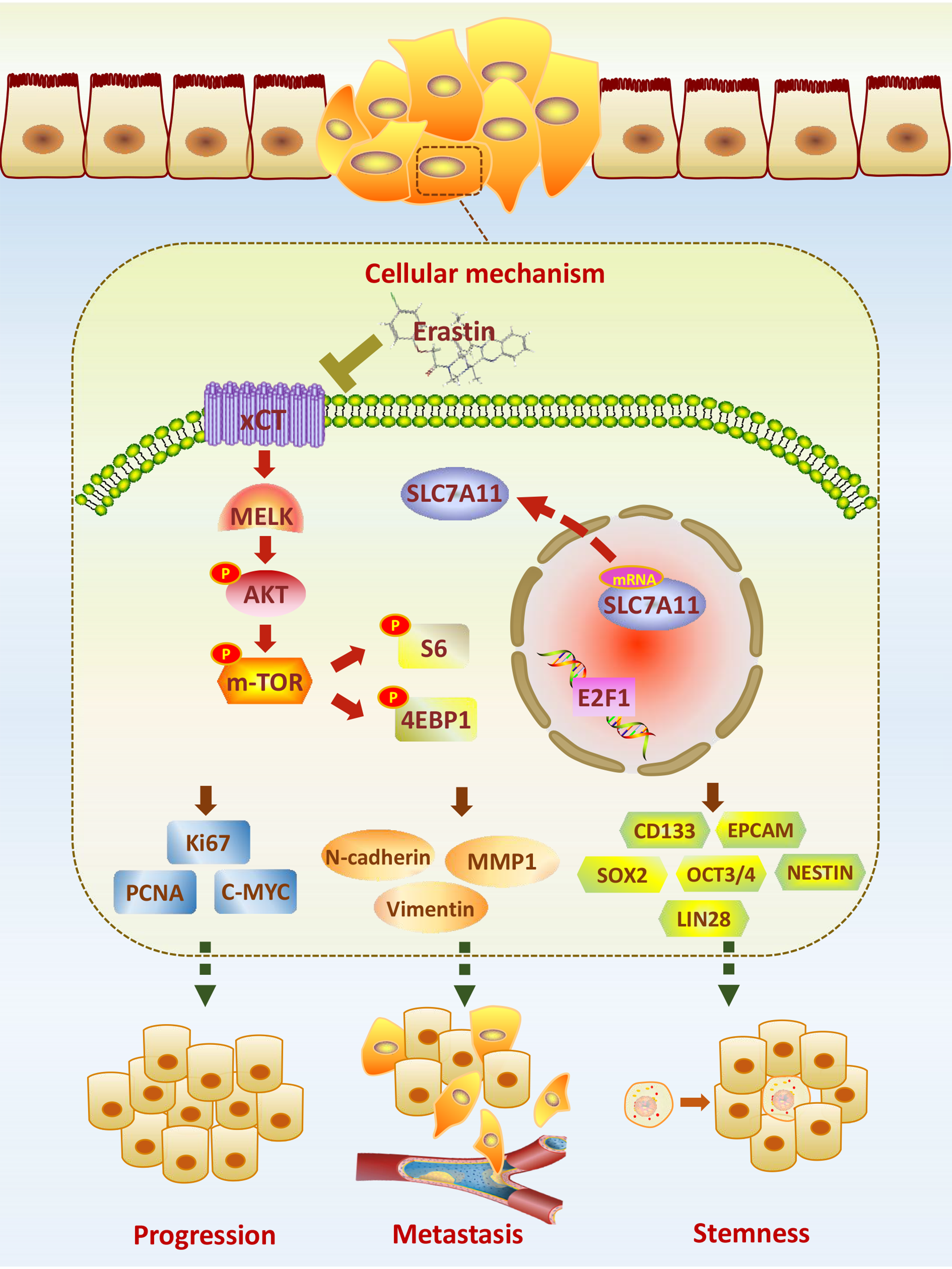

Supplement: Supplementary file 3 — FigureS1 [file 41419_2022_4827_MOESM3_ESM.tif]

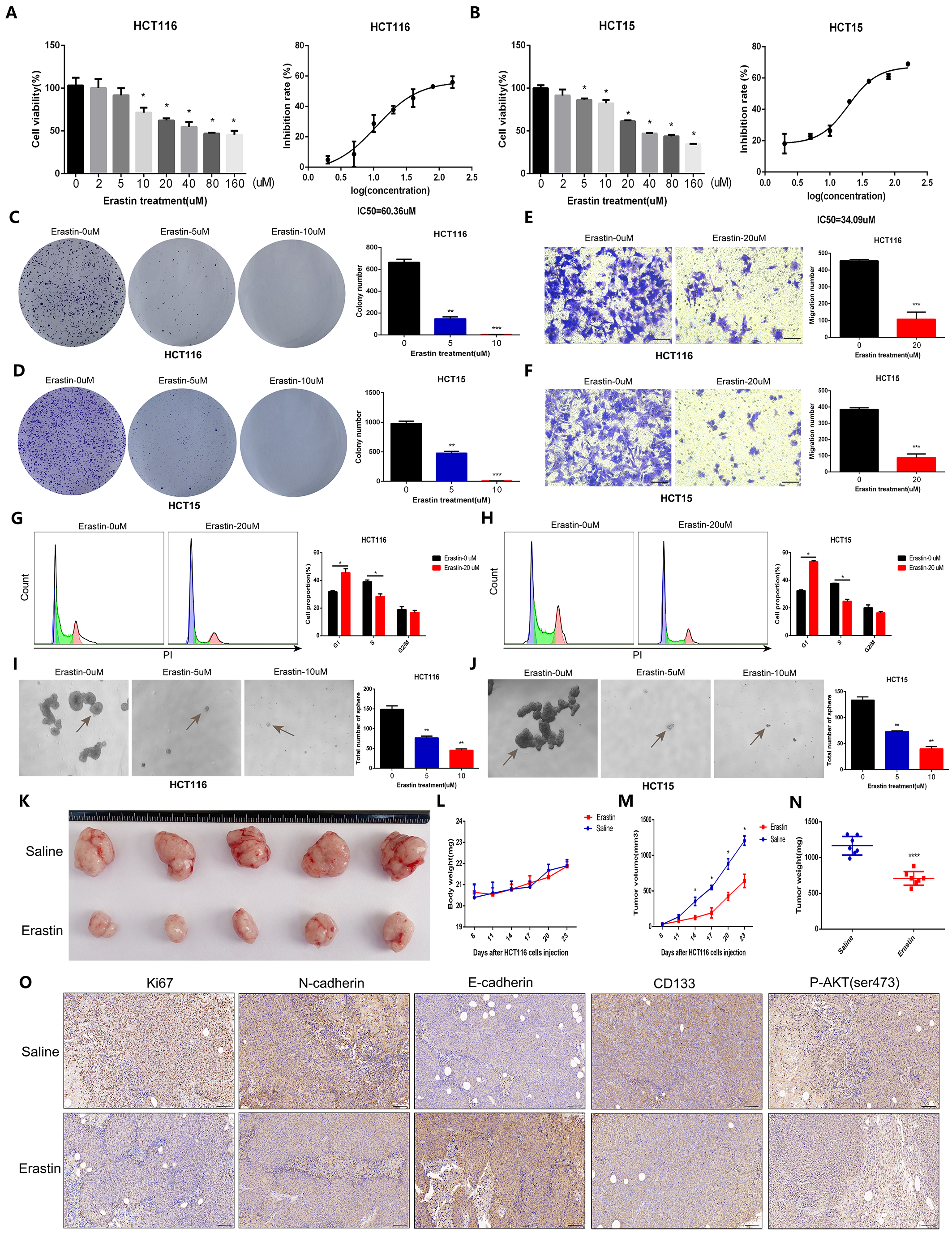

Supplement: Supplementary file 4 — FigureS2 [file 41419_2022_4827_MOESM4_ESM.tif]

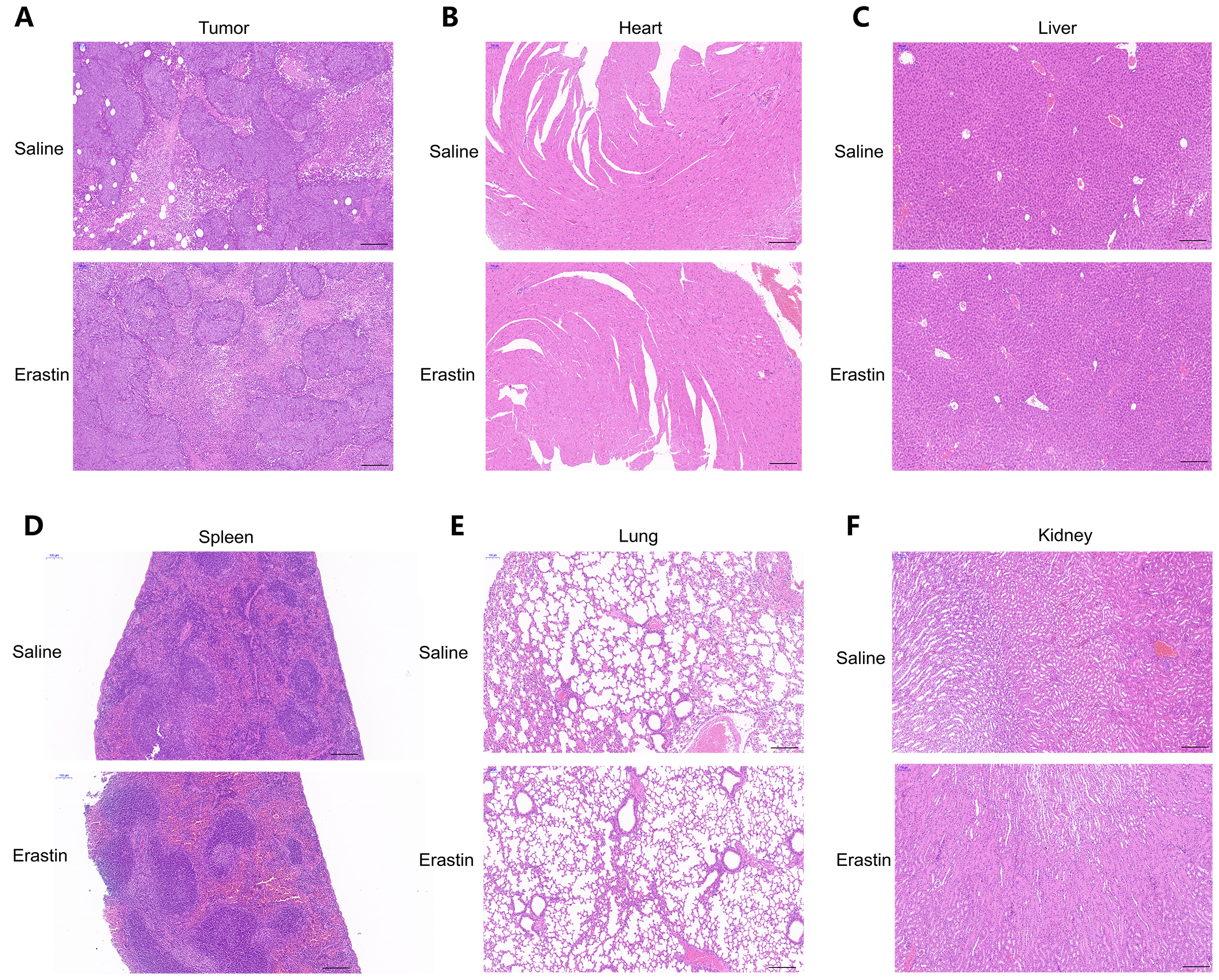

Supplement: Supplementary file 5 — FigureS3 [file 41419_2022_4827_MOESM5_ESM.tif]
