# Supplementary material for: xCT contributes to colorectal cancer tumorigenesis through upregulation of the MELK oncogene and activation of the AKT/mTOR cascade
Source: Cell Death Dis. 2022 Apr 19;13(4):373. doi: 10.1038/s41419-022-04827-4 (PMC9019093; doi:10.1038/s41419-022-04827-4)
Supplement: Supplementary file 6 — supporting information [file 41419_2022_4827_MOESM6_ESM.doc]

**1.1 Sequence based reagents**

| Name |  | Sequence | Supplier |
| --- | --- | --- | --- |
| m β-Actin | Forward | TGAGCTGCGTTTTACACCCT | TSINGKE Biological Technology, Beijing, China. |
|  | Reverse | GCCTTCACCGTTCCAGTTTT |  |
| m xCT | Forward | TACGAAAGTCGCCAGGTCTCT |  |
|  | Reverse | TTCCACCCAGACTCGAACAA |  |
| h xCT | Forward | ATGGGACAAGAAACCCAGGTG |  |
|  | Reverse | TCCCTATTTTGTGTCTCCCCTTG |  |
| h E2F1 | Forward | CCGGGGAATGAAGGTGAACA |  |
|  | Reverse | GAGCAAAAGGGCCGAAAGTG |  |
| h β-actin | Forward | ATCGTCCACCGCAAATGCTTCTA |  |
|  | Reverse | AGCCATGCCAATCTCATCTTGTT |  |

**1.2 Sequence of siRNA and shRNA**

| Name |  | Sequence | Supplier |
| --- | --- | --- | --- |
| xCT | siRNA-1 | CGGCAAACUUAUUGGGUCU | GenePharma, Shanghai, China |
|  | siRNA-2 | AGGGUUAACAAGAGUAUAA |  |
| xCT | shRNA-1 | CCTGCGTATTATCTCTTTATT |  |
|  | shRNA-2 | CCTGTCACTATTTGGAGCTTT |  |
